# The effect of disease misclassification on the ability to detect a gene-environment interaction: implications of the specificity of case definitions for research on Gulf War illness

**DOI:** 10.1186/s12874-023-02092-3

**Published:** 2023-11-20

**Authors:** Robert W. Haley, Jill A. Dever, Gerald Kramer, John F. Teiber

**Affiliations:** 1 Department of Internal Medicine, University of Texas Southwestern Medical Center, Dallas, TX USA; 2 Peter O’Donnell Jr School of Public Health, University of Texas Southwestern Medical Center, Dallas, TX USA; 3 RTI International, Washington, DC USA

**Keywords:** Persian Gulf syndrome, Epidemiologic methods, Research design, Sensitivity and specificity, Statistical power, Environmental exposure, Gene-environment interaction, Surveys and questionnaires

## Abstract

**Background:**

Since 1997, research on Gulf War illness (GWI) has predominantly used 3 case definitions—the original Research definition, the CDC definition, and modifications of the Kansas definition—but they have not been compared against an objective standard.

**Methods:**

All 3 case definitions were measured in the U.S. Military Health Survey by a computer-assisted telephone interview in a random sample (*n* = 6,497) of the 1991 deployed U.S. military force. The interview asked whether participants had heard nerve agent alarms during the conflict. A random subsample (*n* = 1,698) provided DNA for genotyping the *PON1* Q192R polymorphism.

**Results:**

The CDC and the Modified Kansas definition without exclusions were satisfied by 41.7% and 39.0% of the deployed force, respectively, and were highly overlapping. The Research definition, a subset of the others, was satisfied by 13.6%. The majority of veterans meeting CDC and Modified Kansas endorsed fewer and milder symptoms; whereas, those meeting Research endorsed more symptoms of greater severity. The group meeting Research was more highly enriched with the *PON1* 192R risk allele than those meeting CDC and Modified Kansas, and Research had twice the power to detect the previously described gene-environment interaction between hearing alarms and RR homozygosity (adjusted relative excess risk due to interaction [aRERI] = 7.69; 95% CI 2.71–19.13) than CDC (aRERI = 2.92; 95% CI 0.96–6.38) or Modified Kansas without exclusions (aRERI = 3.84; 95% CI 1.30–8.52) or with exclusions (aRERI = 3.42; 95% CI 1.20–7.56). The lower power of CDC and Modified Kansas relative to Research was due to greater false-positive disease misclassification from lower diagnostic specificity.

**Conclusions:**

The original Research case definition had greater statistical power to detect a genetic predisposition to GWI. Its greater specificity favors its use in hypothesis-driven research; whereas, the greater sensitivity of the others favor their use in clinical screening for application of future diagnostic biomarkers and clinical care.

**Supplementary Information:**

The online version contains supplementary material available at 10.1186/s12874-023-02092-3.

## Background

Gulf War illness (GWI) is an often-disabling condition with diverse symptoms such as chronic fatigue, cognitive dysfunction, pain, diarrhea and balance disturbance. It began as an explosive epidemic affecting tens of thousands of deployed U.S. and Coalition military personnel during and immediately after the 6-week Conflict period of the 1991 Persian Gulf War [1]. Initial epidemiologic investigations listed symptoms and potentially toxic environmental exposures [2] but, finding no objective signs or clinical tests to define the condition, were unable to link exposures with the disease [3]. In 1994 Haley et al. used a 2-stage principal components analysis of 52 symptom scales in a study of 249 deployed members of a U.S. Naval Reserve construction battalion to derive the first case definition of GWI including 3 primary variants [4]. The Research case definition was found to be strongly associated with measures of several environmental exposures including low-level organophosphate nerve agent [5]. A series of follow-up clinical case–control studies found associations of the case definition with objective neurophysiologic, autonomic and brain imaging abnormalities [6–9] as well as with a possible genetic marker, the *PON1* Q192R polymorphism, where having the R allele increases susceptibility to nerve agent neurotoxicity [10].

In 1998 Fukuda et al. from the U.S. Centers for Disease Control and Prevention (CDC) described a simpler case definition more amenable to use in large field studies [11]. Later known as the “CDC definition,” a positive result required endorsement of at least 2 of 10 typical GWI symptoms, and a “CDC Severe” subgroup was indicated if the positive symptoms were self-rated as “severe.” Similarly, in 2000 Steele employed a simple case definition, similar to the CDC definition, later after several changes called the “Modified Kansas definition,” which required endorsement of at least 3 of 32 typical symptoms and excluded veterans with any of 10 comorbid conditions [12].

Two later studies applied structural equation modeling to validate the original Research case definition [13, 14], but the CDC and Modified Kansas definitions were never validated. Subsequently additional investigators developed their own case definitions but the original Research, CDC and Modified Kansas definitions became predominant in GWI research.

In 2013 the U.S. Department of Veterans Affairs commissioned a literature review by an ad hoc committee of the Institute of Medicine to propose a standardized case definition [15]. Finding no objective criteria on which to compare the existing case definitions, the committee recommended use of the CDC or Kansas definitions because they were judged to best cover the symptoms most commonly reported by ill Gulf War veterans. Recently, however, the U.S. Military Health Survey (USMHS) reported from a large nationally representative sample of Gulf War veterans a strong association of the original Research case definition with a gene-environment (GxE) interaction of the *PON1* Q192R polymorphism and veterans’ reports of having heard nerve agent alarms in the war. Finding strong evidence of a mechanistic interaction that could not be explained away by errors in measurement, the GxE interaction provided strong evidence of a causal role of low-level sarin in GWI [16, 17].

Since the USMHS collected all 3 case definitions, we reanalyzed the data to compare the GWI symptom profiles of the 3 case definitions and their power to detect the *PON1* Q192R GxE interaction. The findings are relevant to choosing the best uses for each case definition.

## Methods

### Case definitions

The characteristics of the 3 major case definitions are compared in detail in Table S1. The Research case definition dealt with ambiguities in the terms veterans typically used to describe the symptoms by following up each symptom question with 4 to 20 clarifying questions. For example, those who endorsed cutaneous tingling or numbness were asked 15 follow-up questions to describe its anatomical distribution. Then a first-stage principal components factor analysis of the follow-up items generated 3 latent factors and 3 ordinal symptom scales measuring tingling or numbness of 1) the extremities; 2) the face, tongue and lips; and 3) the truck and groin. Thus, the 27 ambiguous symptom questions were parsed into 52 unambiguous symptom scales (Table S2). These symptom scales were then analyzed by a second-stage principal components factor analysis, which identified 6 latent syndrome factors expressed as ordinal syndrome factor scales, the first 3 of which were strong latent factors and other 3 were weak (Fig. S1). These 6 syndrome scales were dichotomized at 1.5 standard deviations above the mean to form binary syndrome indicators (Fig. S2). Veterans positive on any of the 6 indicators met the definition of GWI. Subsequently the 3 strong latent syndrome indicators were used in clinical studies to describe important variations in severity and response to objective measures of pathology [6–10].

The CDC and Modified Kansas case definitions used the raw symptom endorsements qualified by having been present for at least 6 months and classified by a severity rating of mild, moderate or severe (Table S2). The CDC definition was developed in a study of 4 Air Force Reserve units many of whose members had served in the war and remained well enough to serve in the Reserves [11]. Ten typical symptoms, classified in 3 domains—fatigue, mood or cognition, and musculoskeletal pain—were analyzed, and the GWI definition was satisfied by endorsement of at least 1 symptom each from at least 2 of the 3 domains. The subgroup “CDC Severe” was composed of those whose positive symptoms were self-rated as severe. Although the CDC team analyzed 35 symptoms, largely identical to those used for the Research and Modified Kansas definitions, they used only 10 of them to calculate the case definition. The Modified Kansas definition was developed in a large telephone survey of a population-representative sample of Gulf War-era veterans from the state of Kansas which employed a simple case definition, similar to the CDC definition. The “Modified Kansas definition” collected endorsements of 32 symptoms in 6 domains and defined GWI as endorsement of ≥ 2 mild symptoms or ≥ 1 moderate or severe symptom from any 3 of the 6 domains (Table S2) [12].

### National survey of Gulf War veterans

The human subjects who participated in this study were selected from participants of the USMHS, a computer-assisted telephone interview (CATI) of a stratified random sample of military veterans in the U.S. Armed Forces during the 1991 Persian Gulf War conducted from 2007 to 2010. The U.S. Armed Forces personnel list (Defense Manpower Data Center, Seaside, CA) was stratified by the official designations of age, sex, race/ethnicity, military rank, military component (active duty or Reserve/Guard), Kuwaiti Theater of Operations (KTO) deployment (deployed, non-deployed), unit location in KTO on 20 January 1991 (relevant to the deployed only), and special studies strata, and a sample was drawn randomly from the strata. With 74.9% of the selected veterans located and contacted and 80.2% of these agreeing to participate, the overall response rate was 60.1%. Of the full USMHS sample of 8,021 veterans interviewed, 6,497 were deployed to the KTO, and 1,523 were non-deployed. A detailed description of the survey methods and findings has been published [14].

The study protocol, CATI questionnaire and interview script were approved by the institutional review boards of the University of Texas Southwestern Medical Center and RTI International. All participants gave verbal informed consent at the start of the interview and written informed consent before providing a blood sample for DNA.

### Formulation of the GWI case definitions

The CATI questionnaire included the 27 symptoms and 220 follow-up items required to generate the Research case definition [4]; the 10 symptom questions and severity ratings to generate the CDC and CDC Severe case definitions [11]; and 28 of the 32 symptom questions and severity ratings to generate a modified version of the Kansas case definition [12], one version formulated by excluding veterans with cancer, diabetes, heart disease, liver disease, multiple sclerosis, bipolar disorder and schizophrenia (Modified Kansas with exclusions) and another version not making these exclusions (Modified Kansas without exclusions) (Tables S1 and S2). To limit interview length, questions on joint stiffness, generalized body pain, heat and cold sensitivity, and other skin problems were omitted because of overlap with questions on joint and muscle pain, fever and night sweats, and skin rashes. Scoring algorithms described in the published descriptions were followed to formulate the case definitions [4, 11, 12].

### Measures of health-related quality of life

The CATI questionnaire included the 12 standardized questions comprising the Medical Outcomes Study (MOS) Short Form-12 (SF-12), a brief measure of self-reported health-related quality of life that estimates disease burden across diverse health conditions and populations [18]. The SF-12 was developed from the MOS 36-item Short-Form Health Survey SF-36, one of the most widely used instruments for assessing health-related quality of life, validated to reproduce the SF-36 Physical Component Summary and Mental Component Summary scales in the general U.S. population but with fewer questions suitable for high volume surveys or for subjects with short attention spans [19]. The SF-12 scales were normed to 1990 U.S. population means of 60.76 for the Mental and 56.58 for the Physical Summary Scores and standard deviation of 10, with higher scores indicating better health status.

### Measure of low-level nerve agent exposure

To represent individual-level exposure to low-level nerve agent aerosolized by Coalition bombing of Iraqi chemical weapon production and storage sites early in the air campaign phase of the war [20], the following survey question was included in the USMHS CATI: “During the time period from August 2, 1990, to July 31, 1991, did the alarms on the chemical warfare detection devices in areas where you were living or working ever go off while you were present there?”.

### Prevalence case–control sample

A second-stage sample for this study included all CATI participants who met the Research and Modified Kansas definitions and, because of the large numbers involved, a random sample of those meeting the CDC case definition and those who met none of the definitions. These were asked to provide a blood sample for DNA [17]. Of the 2,971 deployed veterans invited, 1,698 (57%) participated, including 1,190 GWI cases meeting any of the GWI case definitions and 508 unaffected controls. A detailed diagram of the sampling plan and participation has been published [17].

### Blood collection and genotyping

Licensed phlebotomists visited the participants in or near their homes and shipped the blood samples overnight on blue ice to the study laboratory where serum and plasma were aliquoted and leukocytes processed for DNA, all of which were frozen at -80°C. Later, the *PON1* Q192R genotype was determined by RT-PCR in triplicate, each individual assay including positive and negative control samples [17].

### Statistical methods

To study the frequency distribution of the number and severity of symptoms endorsed by veterans classified as having GWI by the various case definitions, we formulated a number/severity score for each veteran by weighting each Modified Kansas symptom response as 0 for absent, 1 for mild in severity, and 2 for moderate or severe and summing these scores over the 28 Modified Kansas questions. The distribution of scores was plotted separately for veterans meeting each of the case definitions, and distribution lines were smoothed by the 2-dimensional first degree polynomial negative exponential algorithm using a gaussian weighting kernel in SigmaPlot (Systat Software, San Jose, CA).

Analyses to estimate the population prevalence of GWI by case definition from the full deployed sample of the USMHS (*n* = 6,497) were weighted with the USMHS survey weights to adjust for the unequal probabilities of selection from the strata and selection biases from inability to locate and refusal to participate as described previously [14, 17, 21]. Adjusted standard errors (SEs), allowing for the complex USMHS sampling design, were performed with SAS survey procedures, version 9.4 (SAS Institute, Cary, NC). Potential confounding variables controlled for included age, sex, service branch, rank, active duty/reserve status, special studies strata, and combat exposure level.

The analyses of the GxE interaction were carried out to conform with the recommendations of Knol and VanderWeele [22] for displaying the results of interactions in genetic epidemiologic studies in the familiar 4 × 2 table with a single reference category, which extended the earlier STROBE recommendations [23]. We provided the final measures of interaction on both the additive scale with the relative excess risk due to interaction (RERI) and the multiplicative scale by the prevalence odds ratio (POR) from the interaction term of logistic regression. Both were provided with and without adjustment for confounding. We calculated RERI and its 95% confidence intervals with Zou’s SAS macro [24], which we modified by adding the front end of the Li and Chambless macro [25] to automate the interface with the logistic regression output. Zou’s asymmetric confidence intervals [24] are more accurate than the symmetric ones of Hosmer and Lemeshow and others [26]. Statistical computing was performed with SAS version 9.4 (SAS Institute, Cary, NC).

We assessed the power of the alternative case definitions with the aRERI for the GxE interaction of the PON1 RR vs QQ genotypes and hearing nerve agent alarms. Since the cases meeting the Research definition were a close subset of the highly overlapping groups meeting the CDC and Modified Kansas definitions and thus were the cases on which all 3 definitions agreed, we also calculated the aRERIs for the large subgroups meeting the CDC or Modified Kansas definitions but not the Research definition to assess the power provided by these additional cases. We tested for heterogeneity of the GxE interactions across age and sex strata by tabulating the stratum-specific RERI and with 3-variable logistic regression analyses [27].

### Estimation of sensitivity and specificity of the case definitions

In the absence of a “gold standard” diagnostic test, we adopted the GxE interaction of the *PON1* Q192R genotype and hearing nerve gas alarms measured by the RERI [17] as the objective foundation needed to estimate the true specificity of case definitions. We could readily calculate directly from the database the crude RERI for each case definition which is biased by the case definition’s inherent level of disease misclassification, i.e., the “biased RERI.” The bias in the RERI comes from misclassification that mixes true cases, which have a high probability of exposure, with non-cases, which have a lower probability of exposure, thus reducing the strength of the exposure odds ratio and, in turn, the RERI. In a sensitivity analysis we then used lessons from the rich literature on disease misclassification [28, 29] to recalculate the RERI after correcting the number of exposed and unexposed cases for different levels of specificity of the case definition until the corrected RERI equaled the biased RERI. This identified the true specificity of the case definition.

Adapting the model of Brenner and Savitz to our study design [29], we developed a set of equations to correct the 8 cells of the 4 × 2 table used to calculate the GxE interaction (cases vs controls by exposure, stratified by genotype QQ vs RR) for disease misclassification. We applied these corrections in a series of sensitivity analyses to assess the impact of case definition specificity on the RERI. We then built a spreadsheet to estimate the sensitivity values of the case definitions using their specificities, case and control sample sizes, and population prevalence rates. The set of equations to correct each of the 2 × 2 tables is as follows:

1.1 $$a'=\left({G_i\bullet{Sp}_i\bullet p}_R\right)+\left[{G_i\bullet\left(1-{Sp}_i\right)\bullet p}_C\right]$$

1.2 $$b'=\lbrack G_i\bullet{Sp}_i\bullet\left(1-p_R\right)\rbrack+\left[G_i\bullet\left(1-{Sp}_i\right)\bullet{(1-p}_C)\right]$$

1.3 $$c'=N_C\bullet p_C$$

1.4 $$d'=N_C\bullet(1-p_C)$$

where:

*G*_*i*_ is the number of subjects classified as GWI cases by the *i-th* case definition.

*N*_*C*_ is the number of subjects in the common control group.

*p*_*R*_ is the prevalence rate of sarin exposure in true cases, estimated by the exposure rate in cases from the Research case definition, which is assumed to have perfect specificity.

*P*_*C*_ is the prevalence rate of sarin exposure in true non-cases, estimated by the exposure rate in subjects in the common control group.

*Sp*_*i*_ is the specificity of the *i-th* case definition.

The sensitivity analysis proceeded from the following evidence-based assumptions:

1. The CDC and the Modified Kansas without exclusions case definitions have perfect sensitivity (*Se* = 1.0), while their specificity is to be estimated.
2. The Research case definition has perfect specificity (*Sp* = 1.0), while its sensitivity is to be estimated.
3. If the specificity of a case definition is perfect as we assume for the Research case definition, variations in its sensitivity do not bias the odds ratios of the 2 × 2 tables [29] or the RERI from the genotype-stratified analysis.
4. The control group is composed of all subjects in the study sample not classified as cases by any of the case definitions, so that all case definitions are compared with the same group of controls. Since the CDC and Modified Kansas without exclusions case definitions are assumed to have perfect sensitivity, the control group is assumed to contain no true cases, and thus the size of the control group does not vary with correction for misclassification, unlike the conditions of the Brenner and Savitz model [29].

## Results

### Overlap of the case definitions

**Fig. 1:**
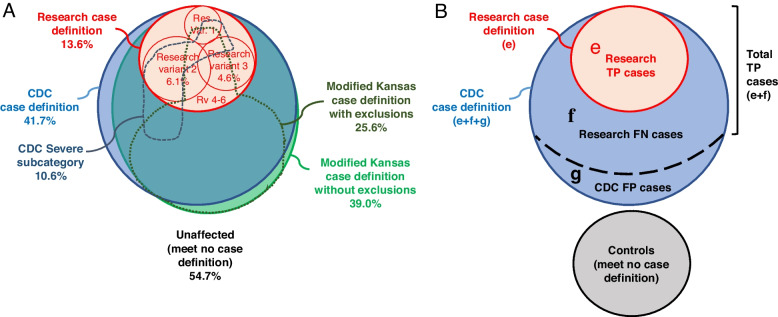
Venn diagrams showing the overlap of GWI case definitions. **A** Overlap of the 3 most used GWI case definitions and their subtypes and variants. The various areas are approximately proportional to their estimated prevalence in the Gulf War-deployed U.S. military population, estimated by applying survey weights to the USMHS deployed sample data (*n* = 6,497) and quantified by the given percentages. **B** Overlap of the Research and CDC case definitions showing that the cases identified by the CDC case definition are composed of 3 zones: true positive cases where Research and CDC agree (**e**), true positive cases missed by Research (Research false negative cases) (**f**), and CDC false positive cases (**g**). Note that as the number of CDC false positive cases increases, the number of Research false negative cases decreases. The number of controls was constant and did not change with corrections for disease misclassification. The Modified Kansas without exclusions case definition (not pictured) is interchangeable with the CDC case definition in (**B**). Abbreviations: FN, false negative; FP, false positive; TP, true positive

Projecting the USMHS sample data to the full deployed force, we found that the CDC case definition and the Modified Kansas case definition with no exclusions, which can be satisfied by a veterans’ having only 2 or 3 symptoms, respectively, were satisfied by large segments (41.7 ± SE 1.3% and 39.0 ± 1.3%, respectively) of the deployed force that overlapped extensively (Fig. 1A; Table S4). The original Research case definition—which is subclassified into 6 variants—was met by a much smaller segment of the deployed force (13.6 ± 0.9%) and was a close subset of the those satisfying the CDC and Modified Kansas definition with no exclusions (Fig. 1A).

The CDC Severe was met by the smallest segment of the deployed force (10.6 ± SE 1.0%) and included one-fourth of those who satisfied the CDC definition and approximately one-third of those meeting the Research case definition (Fig. 1A).

The Modified Kansas case definition with exclusions was satisfied by 25.6 ± SE 1.2% of the deployed force, but the exclusions disproportionately eliminated veterans satisfying the Research case definition and the CDC Severe case definition (Fig. 1A).

The prevalence of the deployed veterans who satisfied the various case definitions and the regions of the Venn diagram defined by their overlap as well as their distributions by the background characteristics are given in Table S4.

### Number/severity of symptom questions endorsed

**Fig. 2:**
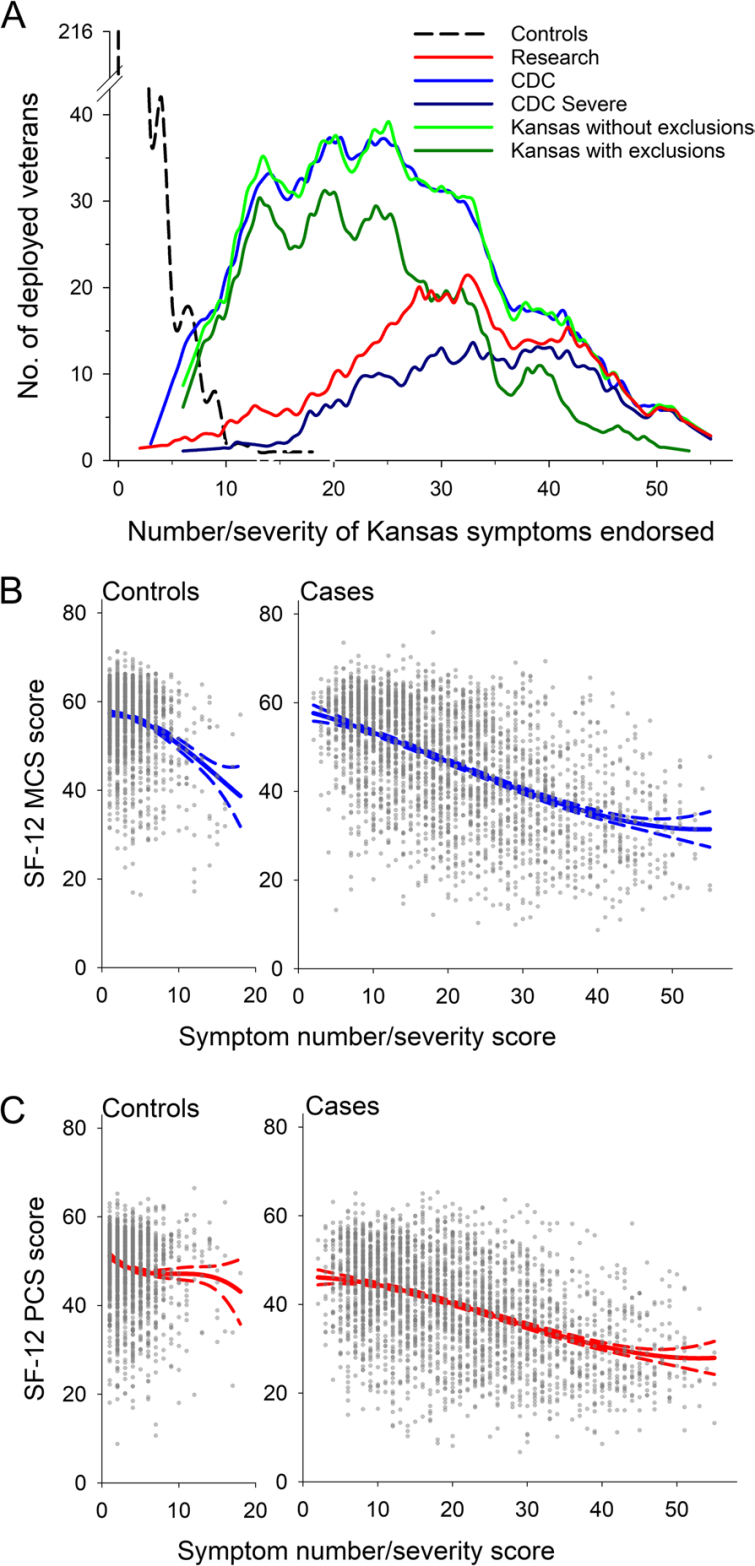
Association of the GWI number/severity score with case definitions and health-related quality of life. **A** Frequency distribution of the GWI number/severity score of 6,497 USMHS deployed veterans meeting the various GWI case definitions. Each line represents the score’s distribution in veterans classified as having GWI by one of the 5 case definitions and the control group of veterans who satisfied none of the case definitions. **B**-**C**, Association of the GWI number/ severity score with the SF-12 Mental Component Summary scores (*r* = -0.054 ± 0.002) (**B**) and Physical Component Summary scores (*r* = -0.064 ± 0.002) (**C**). The GWI number/severity score is an overall measure of the symptoms used to generate the Modified Kansas case definitions. Veterans rated each of the symptoms as absent (0), mild (1), or moderate or severe (2), and a veteran’s number/severity score was formed by summing these ratings over all the symptoms. A score of 0 on an SF-12 summary scale indicates the lowest health-related quality of life, while 100 indicates the highest. Associations of individual case definitions with the SF-12 scores are given in Figure S3 and Table S5

The number/severity score of symptom responses varied among veterans from 0 (endorsed none of the 28 symptoms) to 56 (endorsed all 28 symptoms at moderate or greater severity). Most of the unaffected controls had very low scores indicating they endorsed no symptoms or only a few, mostly mild ones; whereas, those meeting the Research definition and the CDC Severe definition had primarily high scores indicating they endorsed many, mostly at moderate or greater severity (Fig. 2A). In contrast, most of those meeting the CDC and Modified Kansas case definitions had low scores indicating they endorsed fewer symptoms, mostly with mild ratings; only a small proportion had high scores, and virtually all of these were the same veterans who also met the Research and CDC Severe case definitions (Fig. 2A). The process of excluding veterans with qualifying comorbid conditions from the Modified Kansas case definition disproportionately eliminated the veterans with higher scores, leaving a higher proportion of those endorsing fewer or mild symptoms.

**Fig. 3:**
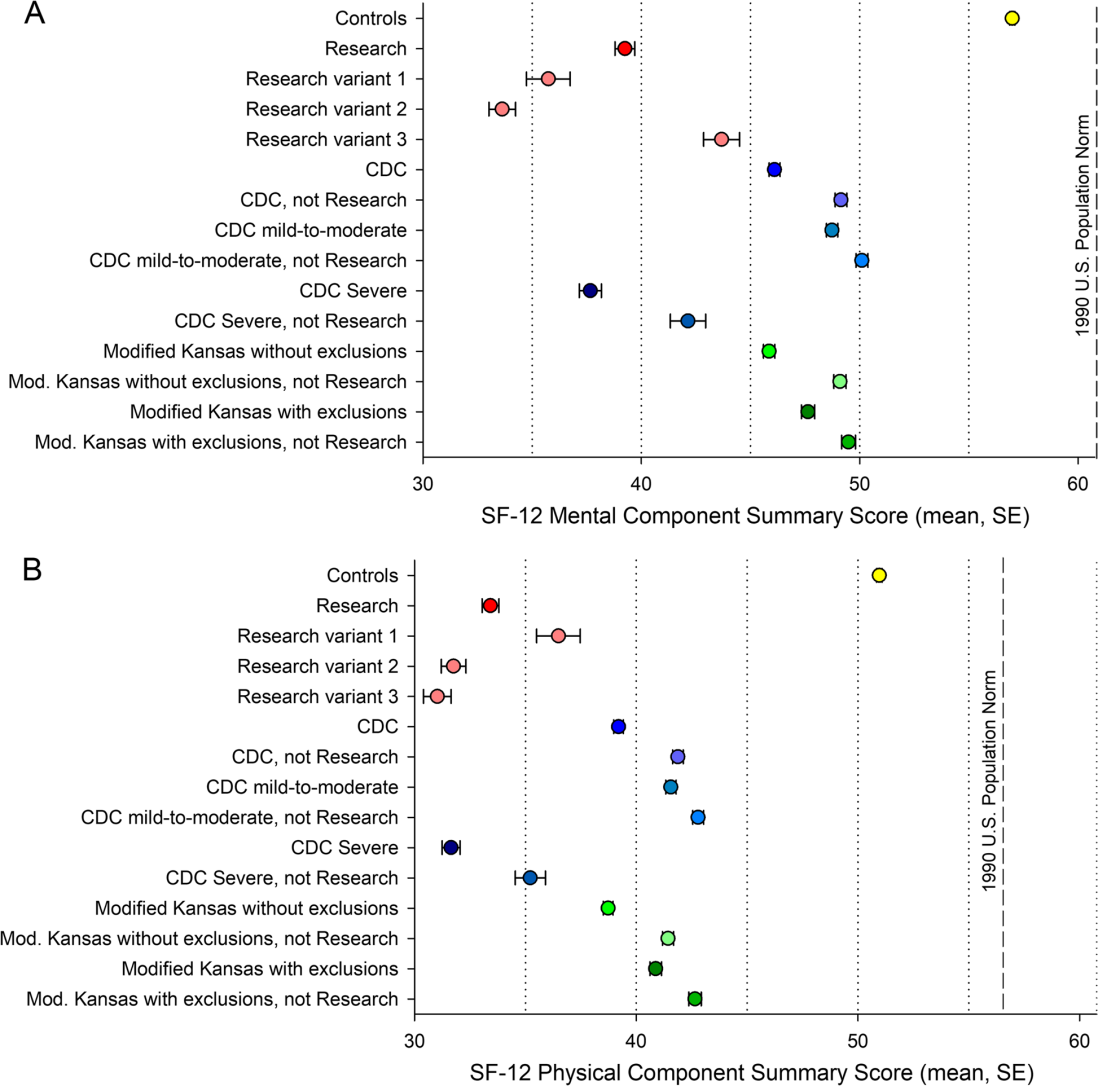
Mean Medical Outcomes Study’s Short Form-12 (SF-12) Mental (**A**) and Physical (**B**) Summary Scores by GWI case definition in the full deployed USMHS sample (*n* = 6,497). The SF-12 scales were normed to 1990 U.S. population means of 60.76 for the Mental and 56.58 for the Physical Summary Scores and standard deviation of 10, with higher scores indicating better health status

The number/severity score was inversely associated with health-related quality of life and disease burden measured by the SF-12 Mental Summary Score and Physical Summary score (Fig. 2B and C; Fig. S3; Table S5). Accordingly, while the control veterans had summary scores at or above the U.S. population mean of 50, veterans meeting the Research and CDC Severe had the lowest summary scores, 1 to 2 standard deviations below those meeting the regular CDC and Modified Kansas definitions (Fig. 3).

### Power to detect an association with nerve agent alarms

**Fig. 4:**
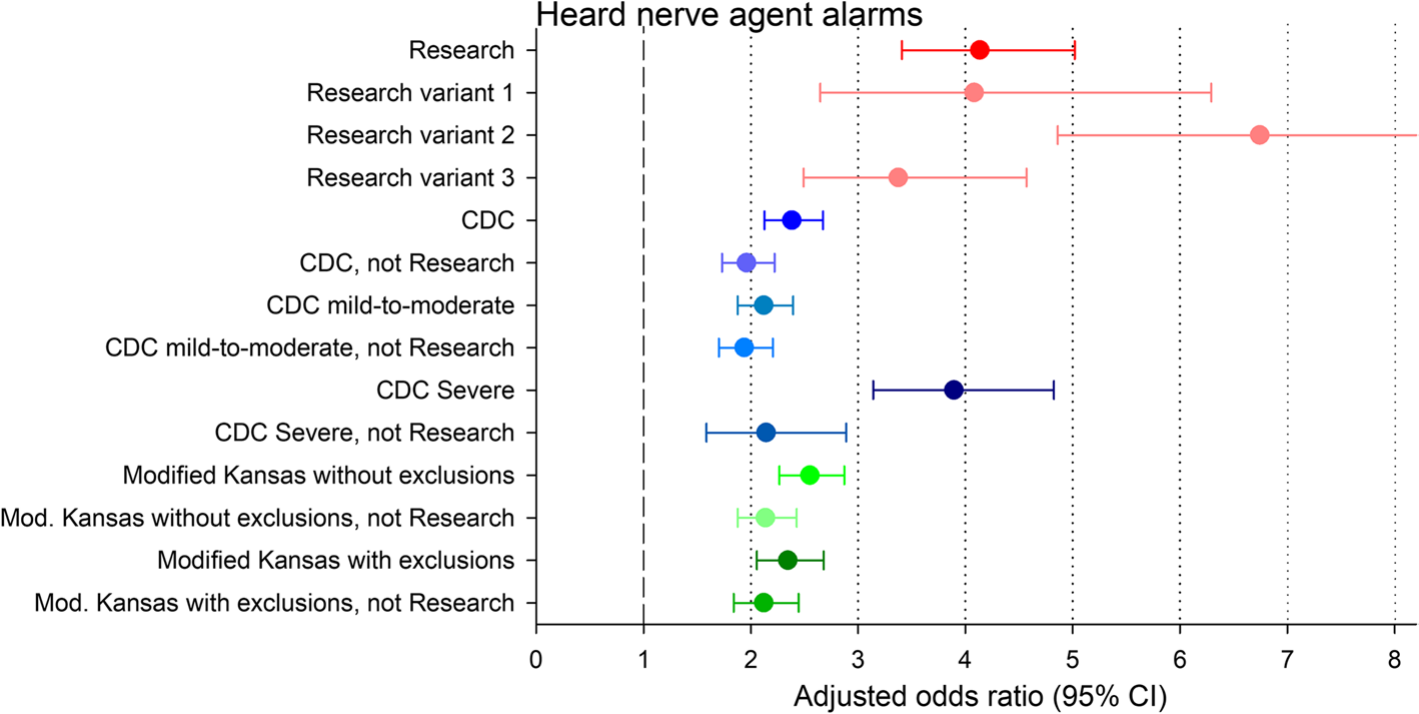
The association of having heard nerve agent alarms in the Gulf War with the various case definitions of Gulf War illness and their overlap, estimated by unweighted logistic regression in the full deployed USMHS sample (*n* = 6,497), adjusted for the confounding variables. Numerical values are given in Table S6

When defined by the Research case definition, GWI was strongly associated with having heard nerve agent alarms in the war (adjusted odds ratio [aOR] 4.12, 95% CI 3.40–5.00), but when it was defined by the CDC or Modified Kansas case definitions, the association was approximately half as strong (Fig. 4; Table S6). Removing the veterans who met the Research definition from the CDC and Modified Kansas definitions further reduced their aORs. The CDC Severe subclassification was the only one with an aOR approximating that of the Research case definition, but removing those meeting the Research case definition from the CDC Severe definition reduced its aOR by half back to the level of the full CDC and Modified Kansas definitions.

### Degree of enrichment in the R allele

**Fig. 5:**
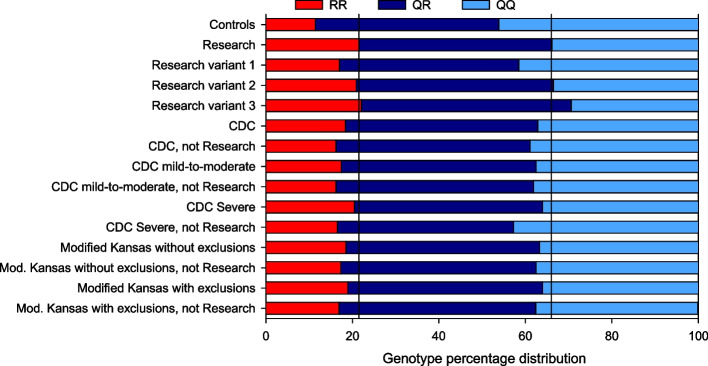
Percentage distribution of the *PON1* Q192R genotype in the USMHS participants who provided DNA including controls and veterans with GWI by the various case definitions and their overlap illustrated in Fig. 1. The data are unadjusted and unweighted. Numerical values and sample sizes are given in Table S7

Compared with the *PON1* Q192R genotype distribution of the control group, that of the group satisfying the Research case definition was enriched for the RR and QR genotypes (i.e., for the R allele) (Fig. 5; Table S7). The groups selected by the CDC and Modified Kansas case definitions were similarly enriched but less so than the group selected by the Research case definition. Removing the group satisfying the Research definition from the CDC and Modified Kansas definitions further reduced their degree of R allele enrichment.

### Power to detect an association with the GxE interaction

Although all of the case definitions detected the association of GWI with the GxE interaction between the *PON1* Q192R polymorphism and hearing nerve agent alarms, the Research case definition provided an aRERI twice the size of those provided by the CDC and Modified Kansas definitions and a greater level of statistical significance despite a far smaller sample size (Table 1; Tables S8-S21). The one exception was that the CDC Severe subcategorization provided an aRERI of approximately the same magnitude as the Research definition; however, like all the CDC and Modified Kansas alternatives, removal of the group meeting the Research definition reduced its aRERI to the lower magnitude of the others.

**Table 1:** Strength of the association of the *PON1* Q192R-nerve agent GxE interaction with the various GWI case definitions

GWI case definition	Number of veterans classified as GWI in case–control sample^a^	aRERI for GxE interaction (95% CI)^b^
Research	508	7.69 (2.71 – 19.13)
Research variant 1	94	3.66 (0.17 – 13.66)
Research variant 2	206	15.84 (5.36 – 56.31)
Research variant 3	163	7.93 (2.38 – 24.16)
CDC	1109	2.92 (0.96 – 6.38)
CDC, not Research^c^	624	1.98 (0.42 – 4.67)
CDC mild-to-moderate	757	2.41 (0.68 – 5.46)
CDC mild-to-moderate, not Research^c^	521	2.08 (0.46 – 4.92)
CDC Severe	352	7.16 (2.34 – 19.18)
CDC Severe, not Research^c^	103	2.11 (-0.85 – 8.95)
Modified Kansas without exclusions	1090	3.84 (1.30 – 8.52)
Modified Kansas without exclusions, not Research^c^	602	2.79 (0.68 – 6.67)
Modified Kansas with exclusions	748	3.42 (1.20 – 7.56)
Modified Kansas with exclusions, not Research^c^	509	2.92 (0.85 – 6.82)

*Abbreviation*: *aRERI* adjusted relative excess risk due to interaction, *CI* confidence interval, *GWI* Gulf War illness, *PON1 Q192R* polymorphism of the *paraoxonase-1* gene

^a^The control group included 508 deployed veterans who satisfied none of these case definitions

^b^The potential confounders controlled for in the adjusted models included: age, sex, service branch, rank, active duty vs Guard/Reserve, special strata, and combat exposure scale. Detailed tables showing the calculation of the aRERI are given in Tables S8-S21

^c^These groups contain those personnel meeting the CDC or Modified Kansas case definitions but excluding those also meeting the Research definition

Although the point estimates of the RERI suggested that the GxE interaction might be stronger in women, we found no statistically significant evidence of heterogeneity by age or sex (Table S22 and S23).

### Sensitivity and specificity of the case definitions

The sensitivity analyses produced by varying the value of specificity in Eqs. 1.1 and 1.2 generated new values of RERI corrected for different levels of disease misclassification (Table 2). When sensitivity and specificity were both perfect, the second term in Eq. 1.1 dropped out, leaving the number of exposed cases ($a'$) in the 2 × 2 tables equal to the total number of diagnosed cases (*G*_*i*_) times the exposure prevalence rate of true cases (*p*_*R*_). The second term in Eq. 1.2 likewise dropped out leaving the number of unexposed cases ($b'$) equal to the total number of diagnosed cases (*G*_*i*_) times 1 minus the exposure prevalence rate of true cases (1-*p*_*R*_). Reducing the value of specificity diluted the exposure prevalence rate of true cases by adding non-cases that had the exposure prevalence rate of controls (*p*_*C*_). As specificity continued to decline, at some point this dilution effect produced the mix of exposure rates present in the original biased data, which we discovered when the corrected RERI equaled the biased RERI. The value of specificity was the case definition’s intrinsic specificity.

**Table 2:** Sensitivity analyses identifying each case definition’s intrinsic specificity from its RERI corrected for disease misclassification

Se	Sp	Corrected RERI By CDC definition		Se	Sp	Corrected RERI By Modified Kansas without exclusions	
1.000	1.000	6.36	(2.50–12.98)	1.000	1.000	6.80	(2.76–13.77)
1.000	0.950	5.69	(2.14–11.74)	1.000	0.950	6.09	(2.37–12.45)
1.000	0.900	5.10	(1.82–10.63)	1.000	0.900	5.45	(2.02–11.27)
1.000	0.850	4.56	(1.53- 9.64)	1.000	0.850	4.88	(1.71–10.22)
1.000	0.830	4.37	(1.42- 9.27)	1.000	0.845	4.83	(1.68–10.12)
1.000	0.820	4.27	(1.37- 9.10)	1.000	0.840	4.77	(1.65–10.02)
**1.000**	**0.819**	**4.26**	(1.36- 9.08)*	**1.000**	**0.839**	**4.76**	(1.64–10.00)*
1.000	0.800	4.09	(1.26- 8.75)	1.000	0.800	4.37	(1.42- 9.27)
1.000	0.700	3.26	(0.80- 7.23)	1.000	0.700	3.48	(0.91- 7.66)
Se	Sp	Corrected RERI By Modified Kansas with exclusions		Se	Sp	Corrected RERI By CDC Severe	
1.000	1.000	6.46	(2.47–13.55)	**1.000**	**1.000**	**7.65**	(2.89–17.15)*
1.000	0.950	5.78	(2.11–12.25)	1.000	0.950	6.85	(2.46–15.47)
1.000	0.900	5.18	(1.78–11.09)	1.000	0.900	6.14	(2.06–13.99)
1.000	0.850	4.64	(1.49–10.06)	1.000	0.850	5.50	(1.70–12.67)
1.000	0.800	4.15	(1.22- 9.14)	1.000	0.800	4.92	(1.37–11.50)
1.000	0.790	4.06	(1.16- 8.96)	1.000	0.750	4.40	(1.06–10.44)
1.000	0.787	4.03	(1.15- 8.91)	1.000	0.700	3.92	(0.78- 9.49)
**1.000**	**0.786**	**4.02**	(1.14- 8.89)*	1.000	0.650	3.48	(0.52- 8.63)
1.000	0.785	4.01	(1.14- 8.88)	1.000	0.600	3.08	(0.28- 7.84)
Se	Sp	Corrected RERI By Research		Se	Sp	Corrected RERI By Research	
**1.000**	**1.000**	**8.88**	(3.77–18.61)*	**1.000**	**1.000**	**8.88**	(3.77–18.61)*
1.000	0.950	7.95	(3.25–16.79)	0.950	1.000	8.88	(3.77–18.61)
1.000	0.900	7.12	(2.77–15.18)	0.900	1.000	8.88	(3.77–18.61)
1.000	0.850	6.38	(2.35–13.75)	0.850	1.000	8.88	(3.77–18.61)
1.000	0.800	5.71	(1.96–12.47)	0.800	1.000	8.88	(3.77–18.61)
1.000	0.750	5.10	(1.60–11.31)	0.750	1.000	8.88	(3.77–18.61)
1.000	0.700	4.55	(1.26–10.27)	0.700	1.000	8.88	(3.77–18.61)
1.000	0.650	4.04	(0.95- 9.32)	0.650	1.000	8.88	(3.77–18.61)
1.000	0.600	3.58	(0.67- 8.46)	0.600	1.000	8.88	(3.77–18.61)

Note that the corrected RERI could not be adjusted for confounding because the corrections for disease misclassification are ecologic, not amenable to multivariable analysis at the subject level; confounding adjustment would reduce these RERIs by 1–2 points

*Abbreviations*: *Se* sensitivity, *Sp* specificity, *RERI* relative excess risk due to interaction

^*^Asterisk indicates the levels of Se and Sp where the corrected RERI equals the biased RERI. Our reference values of biased RERI by case definition are: CDC 4.26 (3.77–18.61); Modified Kansas without exclusions 4.76 (1.64–10.00); Modified Kansas with exclusions 4.02 (1.26–8.69); CDC Severe 7.80 (2.67–18.13); Research 8.88 (3.77–18.61)

This procedure reduced the RERI for the CDC and both Modified Kansas case definitions from their inflated values in the first line of each table where *Se* = 1 and *Sp* = 1 to substantially lower values, identifying their true specificity values substantially below 1 (Table 2). For the Research and CDC Severe case definitions, the uncorrected RERI in the first line of the table equaled the biased RERI, thereby verifying their perfect specificity. Moreover, with the Research case definition having perfect specificity, varying its sensitivity did not alter the corrected RERI (Table 2). The final specificity values for each case definition are given in Table 3.

**Table 3:** Quantitative estimates of the levels and consequences of disease misclassification by GWI case definitions (calculations explained in Table S22)

Case definition	Sensitivity (95% CI)	Specificity (95% CI)	Population prevalence rate of diagnosed cases (%)	Population prevalence rate of true positive cases (%)	Percentage of diagnosed cases that are falsely positive (%)	Percentage of true cases that are falsely negative (%)
CDC	1^a^	0.82 (0.78–0.86)	41.7	34.2	18.1	0
Modified Kansas without exclusions	1^a^	0.84 (0.80–0.88)	39.0	32.7	16.1	0
Modified Kansas with exclusions	0.59 (0.55–0.63)	0.79 (0.74–0.82)	25.6	20.1	21.4	41.1
Research	0.40 (0.36–0.43)	1^b^^,c^	13.6	13.6	0	60.2
CDC Severe	0.31 (0.28–0.35)	1^c^	10.6	10.6	0	69.0

^a^Perfect sensitivity was assumed from cases satisfying the case definition with only 2 or 3 GWI symptoms, respectively

^b^Perfect specificity was assumed from the Research case definition’s requiring high-threshold fit to data-derived GWI symptom patterns developed with principal components analysis of symptom scales

^c^Perfect specificity was found in sensitivity analysis

From the final specificity values, the case and control sample sizes and the population prevalence rates of the case definitions (Fig. 1A), we estimated the sensitivity values and related statistics of the case definitions (Table 3). The Research and CDC Severe case definitions achieved perfect specificity at the expense of low sensitivity: 0.40 (0.36–0.43) and 0.31 (0.28–0.35), respectively. The Modified Kansas with exclusions had the lowest specificity and low sensitivity as well. The spreadsheet used for these calculations is reproduced as Table S24.

## Discussion

The central finding of our study is that, of the 3 commonly used GWI case definitions, the original Research definition had twice the statistical power as the CDC and Modified Kansas definitions for detecting the associations of GWI with having heard nerve agent alarms, the enrichment of the PON1 Q192R polymorphism, and their GxE interaction. This is important because this genetic finding represents the first compelling evidence for an etiology of GWI, and without the Research definition the association would probably not have been discovered. The reason for this difference in statistical power appears related to differences in the stringency of defining a case. Most veterans meeting the Research case definition endorsed larger numbers of symptoms of greater severity associated with substantial impairment in health-related quality of life measured by the SF-12 scores; whereas, those meeting the CDC and Modified Kansas case definitions, though encompassing those meeting the Research definition, included mostly veterans with smaller numbers of milder symptoms associated with higher health-related quality of life with little difference, on average, from that of the control group of subjects not meeting any case definition. Temporarily omitting those participants also meeting the Research definition from the rest meeting the CDC and Modified Kansas-positive cases uniformly reduced the statistical power of those 2 case definitions, confirming that the large number of veterans in the remaining subset contained more misclassified subjects.

This difference in stringency of defining a case is explained by the construction of the case definitions (Table S1). The CDC and Modified Kansas case definitions are satisfied by a veteran’s having as few as 2 or 3 individual symptoms from categories of symptoms commonly found in many conditions in civilian life. Whereas development of the Research definition started with virtually the same list of symptoms (Table S2), it used two-stage principal components factor analysis first to parse each of the ambiguous raw symptom questions into unambiguous symptom scales, and then it used a factor-weighted sum of all the symptom scales to identify reproducible symptom complexes so that an individual veteran had to share a complex of symptoms with other ill veterans and exceed a high threshold on the syndrome scales of these complexes to be classified as a case. The resulting Research case definition presented a much higher threshold to satisfy which was met by only approximately one-third of those who met the CDC and Modified Kansas without exclusions. Consequently, CDC and Modified Kansas definitions are highly inclusive (high sensitivity) but include many non-cases in the case group (low specificity); in contrast, the Research definition selects high probability cases (high specificity) but misses many true cases (low sensitivity). The Research definition detected manifestations of environmental chemical exposures with greater specificity; whereas, by requiring only a few individual symptoms, which occur commonly in other non-war-related conditions, the CDC and Modified Kansas definitions provided greater sensitivity for a wide range of conditions from severe and mild chemical exposures to diverse chronic illnesses and injuries possibly unrelated to deployment but at the expense of lower specificity for GWI.

To directly test this explanation, we developed a method of estimating the diagnostic sensitivity and specificity of each GWI case definition using detection of the GxE interaction in place of a “gold standard” diagnostic test, which does not yet exist. From a review of the extensive literature on disease misclassification in epidemiology [28], we adapted to our study design the mathematical model of Brenner and Savitz for correcting the odds ratio for disease misclassification in case–control studies [29]. Their model assessed the separate and combined effects of sensitivity and specificity to determine which should be maximized in choosing a case definition for a case–control study in which the relative sample sizes of both the case and control groups could vary. In our study design, however, the control group had already been selected to contain no subjects meeting any of the 5 case definitions being compared and thus was static, which simplified our problem. Moreover, the specifications of the case definitions as well as the Venn diagram of their overlaps (Fig. 1) justified the simplifying assumptions that the CDC and Modified Kansas without exclusions had perfect sensitivity while the Research case definition had perfect specificity. With these assumptions our adaptation of the Brenner-Savitz correction equations could make corrections for disease misclassification on the RERI as a function of the specificity of the case definition used. This corrected RERI could then be compared with the biased RERI calculated directly from the study data so that the specificity at which the 2 RERI estimates agree would identify the intrinsic specificity of the case definition.

In support of our hypothesis, under the assumption of perfect sensitivity, the CDC and Modified Kansas without exclusions definitions were found to have reduced specificities of 0.82 (0.78–0.86) and 0.84 (0.80–0.88), respectively. Excluding subjects with comorbid diseases, however, reduced both specificity [0.79 (0.74–0.82)] and sensitivity [0.59 (0.55–0.63)] of the Modified Kansas definition with exclusions. Under the assumption of perfect specificity the Research and CDC Severe definitions had sensitivities of 0.40 (0.36–0.43) and 0.31 (0.28–0.35), respectively (Fig. 1B and Table 3). As Brenner and Savitz established [29], we found that with perfect specificity, even though the reduced sensitivity caused the Research and CDC Severe definitions to miss 60% and 69% of true GWI cases, respectively, this did not affect their power to detect the RERI of the GxE interaction; whereas, the reduced specificity of the CDC and Modified Kansas definitions caused severe losses of power (Tables 2 and 3).

These findings reaffirmed the conclusion of Brenner and Savitz that for research studies case definitions that maximize specificity at the expense of sensitivity, such as Research and CDC Severe, are superior to those that maximize sensitivity over specificity, such as the CDC and Modified Kansas definitions [29]. Consequently, employing a series of diagnostic tests, all of which must be positive to qualify as a case or careful screening of all prospective cases to remove false positives are crucial to maximize specificity. In contrast, case definitions with looser criteria tend to perform better for clinical practice where it is important to maximize the number of ill patients included in treatment, and research hypotheses are not being tested [29].

Our finding of reduced specificity and sensitivity of the Modified Kansas definition with exclusions supports the growing practice of reducing or eliminating the exclusion of comorbidities from the Modified Kansas case definition [30]. Phasing out the exclusions has been prompted by the realization that as veterans age, they acquire more of the age-related comorbidities, either incidentally or as GWI necessitates a sedentary lifestyle [31]. In the original population-based study in Gulf War veterans from Modified Kansas, 34% of Gulf War veterans met the Modified Kansas case definition with exclusions [12], but in our nationwide population-based survey performed 10 years later, only 25.6% now met the criteria after comorbidity exclusions were made. Moreover, our analysis found that the exclusions disproportionately eliminated more severely ill veterans but did not improve specificity or statistical power.

An unexpected finding was that the CDC Severe subgroup [11] had almost as much statistical power as the Research case definition. This was due to its primarily selecting the same subset of ill veterans as the Research definition (Fig. 1A). Ironically, in our literature review we found only two instances where the CDC Severe subclassification was used in a study of GWI [32, 33], although its relative ease of collection suggests it could be in the future. Its use, however, is also limited by the small percentage of GWI cases it selects.

A potential limitation of the study is that, whereas the Research and CDC case definitions were originally designed and applied as self-administered written questionnaires (Kansas was originally administered by telephone), in the present study the information for all 3 case definitions was acquired in the telephone interviews by trained professional interviewers following a computerized script. In adapting the original questionnaires to an interview script, we put the information into a conversational format and omitted 4 of the 32 symptom questions we found duplicative from the Modified Kansas question set as part of a reduction in interview length. While any changes are likely to alter the information obtained, the fact that we embedded the identical wording in the script and the omitted questions were duplicative suggests that the interviews collected largely the same information.

Moreover, over the years the CDC and Modified Kansas question sets have been adapted and applied variously in many contexts [34, 35], and the list of exclusionary conditions has been altered in diverse ways [30], both affecting the information obtained. Consequently, although our interview survey may have introduced some differences from the original applications of these case definitions, we believe that our study well captures the differences in misclassification and power of the alternative approaches to case definition development and use.

These findings have important implications for the selection and use of these case definitions in future GWI research. While all 3 detected the associations with the risk factors, the approximately 50 percent loss of statistical power by the CDC and Modified Kansas case definitions reflects that a high proportion of their cases are falsely positive misclassifications. When misclassified subjects comprise a substantial proportion of total cases, final conclusions can be severely biased [36]. In clinical case–control studies testing for pathophysiologic or diagnostic biomarkers, common in this field, if the misclassification in the GWI diagnosis is nondifferential (i.e., unassociated with the risk factors), then the bias only reduces the power to reject the null hypothesis. In this case avoiding a type II error requires estimating the loss of power in the design phase and increasing the sample size to compensate. If, however, the bias is differential, so that only the cases spuriously diagnosed with GWI are associated with a risk factor, the investigators might falsely conclude that the risk factor is a cause of GWI. Similarly, in a randomized clinical trial of treatment in veterans meeting the CDC or Modified Kansas case definitions of GWI, a current priority of funding agencies, if many patients with mild depression, not severe enough to require hospitalization, are spuriously classified as GWI because they have, say, chronic fatigue, difficulty concentrating and functional pain—common symptoms of depression that might meet both CDC and Modified Kansas case definitions—then a treatment that improves depression but not GWI might be falsely labeled an effective treatment for GWI [36].

To avoid such costly errors, epidemiologic and clinical case–control studies and clinical trials using the CDC or Modified Kansas case definitions should add additional tests to screen out false positives [29], as in a recent study detecting mitochondrial dysfunction in GWI [37]. Alternatively, they should embed sub-studies to estimate the rate of misclassification and then correct for it, a practice that has been extensively recommended but rarely applied [38, 39]. Alternatively, use of a more restrictive case definition such as the original Research case definition or the CDC Severe subclassification, might be preferable [36]. Since GWI prevalences are lower with these, they may incur greater costs in recruitment, but this might be preferable to falsely negative results or spurious conclusions from highly misclassifying case definitions.

Finally, some may struggle to understand why so much is being made over misclassification in the case definition when in the normal practice of epidemiology this is rarely encountered. We believe this is because in most studies the case definition is based on relatively precise measures, such as pathogen identification, diagnostic laboratory tests, etc. This avoids substantial misclassification of non-cases as cases, thus automatically achieving high Sp of the case definition. In the presence of high Sp, not capturing all the true cases (low Se) has no adverse effect on the analysis and conclusions. This is why we routinely collect only a subset of the true cases and non-cases with minimal bias. Only when studying diseases diagnosed by highly imprecise case definitions prone to misclassification of non-cases as cases, such as GWI, does low Sp of the case definition become an issue. Even then, when the low Sp of an imprecise case definition is recognized, it is often intuitively resolved by applying additional classification steps such as a diagnostic interview to weed out misclassified cases [29].

## Conclusions

Our evaluation of the 3 case definitions from a large population-representative sample of Gulf War veterans against an objective standard contradicts the conclusions of the Institute of Medicine’s ad hoc committee [15]. Specifically, the CDC and Modified Kansas case definitions do not cover a more representative set of Gulf War veterans’ symptoms, and their simplistic construction allows greater misclassification of GWI non-cases as cases. The substantially reduced diagnostic specificity generally reduces statistical power and may lead to spurious conclusions. Consequently, the greater specificity of the Research and CDC Severe definitions make them better suited for hypothesis-driven research; whereas, the greater sensitivity of the CDC and Modified Kansas definitions make them better suited for clinical screening. Ideally, all 3 will eventually be supplanted by objective diagnostic biomarkers.

### Supplementary Information

**Additional file 1: Table S1.** Description and comparison of the three most commonly used case definitions of Gulf War illness. **Table S2.** Comparison of symptom measures used by the 3 case definitions to define GWI. **Figure S1.** An unrotated scree plot (top) and a second one after varimax rotation (bottom) generated by the principal components factor analysis of the 52 symptom scales from the 249 Gulf War veterans in the Developmental Sample. **Figure S2.** Distributions of the deployed Gulf War veterans on each of the 6 syndrome factor scales from the original Developmental study in the Naval Reserve Battalion and the 2 validation samples. **Table S3.** Goodness-of-fit validation statistics for structural equation model of Gulf War illness with 3 first-order factors (syndrome variants) and a second-order factor (overall Gulf War illness),a by study and sample within study. **Table S4.** Demographic and military characteristics of the deployed population and the controls and GWI cases by the various definitions and their overlap. **Figure S3.** Mean (SEM) SF-12 Mental and Physical Component Scores by GWI case definition in 6,497 deployed veterans. **Table S5. **Mean SF-12 Mental and Physical Component Scores by GWI case definitions measured in 6,497 deployed Gulf War veterans. **Table S6.** The association of having heard nerve agent alarms in the Gulf War with the various case definitions of Gulf War illness and their overlap, estimated by unweighted logistic regression in the full deployed USMHS sample, adjusted for the confounding variables age, sex, service branch, rank, active duty vs Guard/Reserve, special strata, and combat exposure scale (numerical values for Figure 3). **Table S7.** Percentage distribution of the PON1 Q192R genotype in the unaffected controls and groups of  cases defined by the alternative GWI case definitions in the genetics subsample of the USMHS (numerical values for Figure 4). **Table S8.** Interaction on the additive and multiplicative scales of hearing nerve agent alarms and PON1 Q192R genotype on GWI by the original Research case definition. **Table S9.** Interaction on the additive and multiplicative scales of hearing nerve agent alarms and PON1 Q192R genotype on GWI by the Research Variant 1 case definition. **Table S10.** Interaction on the additive and multiplicative scales of hearing nerve agent alarms and PON1 Q192R genotype on GWI by the Research Variant 2 case definition. **Table S11.** Interaction on the additive and multiplicative scales of hearing nerve agent alarms and PON1 Q192R genotype on GWI by the Research Variant 3 case definition. **Table S12.** Interaction on the additive and multiplicative scales of hearing nerve agent alarms and PON1 Q192R genotype on GWI by the CDC case definition. **Table S13.** Interaction on the additive and multiplicative scales of hearing nerve agent alarms and PON1 Q192R genotype on GWI by the CDC mild-to-moderate case definition. **Table S14.** Interaction on the additive and multiplicative scales of hearing nerve agent alarms and PON1 Q192R genotype on GWI by the CDC Severe case definition. **Table S15.** Interaction on the additive and multiplicative scales of hearing nerve agent alarms and PON1 Q192R genotype on GWI by the Modified Kansas without exclusions case definition. **Table S16.** Interaction on the additive and multiplicative scales of hearing nerve agent alarms and PON1 Q192R genotype on GWI by the Modified Kansas with exclusions case definition. **Table S17.** Interaction on the additive and multiplicative scales of hearing nerve agent alarms and PON1 Q192R genotype on GWI by the CDC case definition excluding those meeting the Research case definition. **Table S18.** Interaction on the additive and multiplicative scales of hearing nerve agent alarms and PON1 Q192R genotype on GWI by the CDC mild-to-moderate case definition excluding those meeting the Research case definition. **Table S19.** Interaction on the additive and multiplicative scales of hearing nerve agent alarms and PON1 Q192R genotype on GWI by the CDC Severe case definition excluding those meeting the Research case definition. **Table S20.** Interaction on the additive and multiplicative scales of hearing nerve agent alarms and PON1 Q192R genotype on GWI by the Modified Kansas with no exclusions case definition excluding those meeting the Research case definition. **Table S21.** Interaction on the additive and multiplicative scales of hearing nerve agent alarms and PON1 Q192R genotype on GWI by the Modified Kansas with exclusions case definition excluding those meeting the Research case definition. **Table S22.** Test for heterogeneity of the GxE interaction over age groups, by GWI case definition controlling for confounding. **Table S23.** Test for heterogeneity of the GxE interaction over sex, by GWI case definition controlling for confounding. **Table S24.** Estimation of sensitivity and specificity of GWI case definitions.

## Data Availability

The data are the property of the U.S. Department of Veterans Affairs and are not publicly available because the records can be linked with identifiable protected health information. They were analyzed for this research under a data use agreement between the VA North Texas Health Care System and the University of Texas Southwestern Medical Center at Dallas as part of IDIQ contract # VA 549P-0027.

## Abbreviations

aOR: Adjusted odds ratio
aRERI: Adjusted relative excess risk due to interaction
CATI: Computer-assisted telephone interview
CDC: Centers for Disease Control and Prevention
DNA: Deoxyribonucleic acid
GWI: Gulf War illness
GxE: Gene-environment interaction
KTO: Kuwaiti Theater of Operations
PON1: Paraoxonase-1
POR: Prevalence odds ratio
RT-PCR: Reverse transcriptase polymerase chain reaction
SAS: Statistical Analysis System
Se: Sensitivity
SF-12: 12-Question Short Form of the SF-36
Sp: Specificity
USMHS: U.S. Military Health Survey
