# Supplementary material for: The effect of disease misclassification on the ability to detect a gene-environment interaction: implications of the specificity of case definitions for research on Gulf War illness
Source: BMC Med Res Methodol. 2023 Nov 20;23:273. doi: 10.1186/s12874-023-02092-3 (PMC10659093; doi:10.1186/s12874-023-02092-3)
Supplement: Supplementary file 1 — Additional file 1: Table S1. Description and comparison of the three most commonly used case definitions of Gulf War illness. Table S2. Comparison of symptom measures used by the 3 case definitions to define GWI. Figure S1. An unrotated scree plot (top) and a second one after varimax rotation (bottom) generated by the principal components factor analysis of the 52 symptom scales from the 249 Gulf War veterans in the Developmental Sample. Figure S2. Distributions of the deployed Gulf War veterans on each of the 6 syndrome factor scales from the original Developmental study in the Naval Reserve Battalion and the 2 validation samples. Table S3. Goodness-of-fit validation statistics for structural equation model of Gulf War illness with 3 first-order factors (syndrome variants) and a second-order factor (overall Gulf War illness),a by study and sample within study. Table S4. Demographic and military characteristics of the deployed population and the controls and GWI cases by the various definitions and their overlap. Figure S3. Mean (SEM) SF-12 Mental and Physical Component Scores by GWI case definition in 6,497 deployed veterans. Table S5. Mean SF-12 Mental and Physical Component Scores by GWI case definitions measured in 6,497 deployed Gulf War veterans. Table S6. The association of having heard nerve agent alarms in the Gulf War with the various case definitions of Gulf War illness and their overlap, estimated by unweighted logistic regression in the full deployed USMHS sample, adjusted for the confounding variables age, sex, service branch, rank, active duty vs Guard/Reserve, special strata, and combat exposure scale (numerical values for Figure 3). Table S7. Percentage distribution of the PON1 Q192R genotype in the unaffected controls and groups of cases defined by the alternative GWI case definitions in the genetics subsample of the USMHS (numerical values for Figure 4). Table S8. Interaction on the additive and multiplicative scales of hearing nerve agent alarms [file 12874_2023_2092_MOESM1_ESM.docx]

**Supplementary Digital Content**

The effect of disease misclassification on the ability to detect a gene-environment interaction: implications of the specificity of case definitions for research on Gulf War illness

**Authors:** Robert W. Haley, Jill Dever, Gerald Kramer, John F. Teiber

**Contents**

**Table S1**. Description and comparison of the three most commonly used case definitions of Gulf War illness

**Table S2.** Comparison of symptom measures used by the 3 case definitions to define GWI

**Figure S1.** An unrotated scree plot (top) and a second one after varimax rotation (bottom) generated by the principal components factor analysis of the 52 symptom scales from the 249 Gulf War veterans in the Developmental Sample

**Figure S2.** Distributions of the deployed Gulf War veterans on each of the 6 syndrome factor scales from the original Developmental study in the Naval Reserve Battalion and the 2 validation samples

**Table S3**. Goodness-of-fit validation statistics for structural equation model of Gulf War illness with 3 first-order factors (syndrome variants) and a second-order factor (overall Gulf War illness),^a^ by study and sample within study

**Table S4**. Demographic and military characteristics of the deployed population and the controls and GWI cases by the various definitions and their overlap

**Figure S3.** Mean (SEM) SF-12 Mental and Physical Component Scores by GWI case definition in 6,497 deployed veterans

**Table S5.** Mean SF-12 Mental and Physical Component Scores by GWI case definitions measured in 6,497 deployed Gulf War veterans

**Table S6.** The association of having heard nerve agent alarms in the Gulf War with the various case definitions of Gulf War illness and their overlap, estimated by unweighted logistic regression in the full deployed USMHS sample, adjusted for the confounding variables age, sex, service branch, rank, active duty vs Guard/Reserve, special strata, and combat exposure scale (numerical values for Figure 3)

**Table S7.** Percentage distribution of the *PON1* Q192R genotype in the unaffected controls and groups of cases defined by the alternative GWI case definitions in the genetics subsample of the USMHS (numerical values for Figure 4)

**Table S8**. Interaction on the additive and multiplicative scales of hearing nerve agent alarms and *PON1* Q192R genotype on GWI by the **original Research** case definition

**Table S9**. Interaction on the additive and multiplicative scales of hearing nerve agent alarms and *PON1* Q192R genotype on GWI by the **Research Variant 1** case definition

**Table S10**. Interaction on the additive and multiplicative scales of hearing nerve agent alarms and *PON1* Q192R genotype on GWI by the **Research Variant 2** case definition

**Table S11**. Interaction on the additive and multiplicative scales of hearing nerve agent alarms and *PON1* Q192R genotype on GWI by the **Research Variant 3** case definition

**Table S12**. Interaction on the additive and multiplicative scales of hearing nerve agent alarms and *PON1* Q192R genotype on GWI by the **CDC** case definition

**Table S13**. Interaction on the additive and multiplicative scales of hearing nerve agent alarms and *PON1* Q192R genotype on GWI by the **CDC** **mild-to-moderate** case definition

**Table S14**. Interaction on the additive and multiplicative scales of hearing nerve agent alarms and *PON1* Q192R genotype on GWI by the **CDC Severe** case definition

**Table S15**. Interaction on the additive and multiplicative scales of hearing nerve agent alarms and *PON1* Q192R genotype on GWI by the **Modified Kansas without** **exclusions** case definition

**Table S16**. Interaction on the additive and multiplicative scales of hearing nerve agent alarms and *PON1* Q192R genotype on GWI by the **Modified Kansas with exclusions** case definition

**Table S17.** Interaction on the additive and multiplicative scales of hearing nerve agent alarms and *PON1* Q192R genotype on GWI by the **CDC case definition** **excluding those meeting the Research** case definition

**Table S18**. Interaction on the additive and multiplicative scales of hearing nerve agent alarms and *PON1* Q192R genotype on GWI by the **CDC mild-to-moderate case definition** **excluding those meeting the Research** case definition

**Table S19**. Interaction on the additive and multiplicative scales of hearing nerve agent alarms and *PON1* Q192R genotype on GWI by the **CDC Severe case definition** **excluding those meeting the Research** case definition

**Table S20**. Interaction on the additive and multiplicative scales of hearing nerve agent alarms and *PON1* Q192R genotype on GWI by the **Modified Kansas with no exclusions case definition** **excluding those meeting the Research** case definition

**Table S21**. Interaction on the additive and multiplicative scales of hearing nerve agent alarms and *PON1* Q192R genotype on GWI by the **Modified Kansas with exclusions case definition** **excluding those meeting the Research** case definition

**Table S22**. Test for heterogeneity of the GxE interaction over **age groups,** by GWI case definition controlling for confounding

**Table S23**. Test for heterogeneity of the GxE interaction over **sex,** by GWI case definition controlling for confounding

**Table S24**. Estimation of sensitivity and specificity of GWI case definitions

| **Table S1.** Description and comparison of the three most commonly used case definitions of Gulf War illness | | | |
| --- | --- | --- | --- |
| Case definition | **Original Research case definition** | **CDC case definition** | **Kansas case definition** |
| Reference | Haley et al. 1997 [1] | Fukuda et al. 1998 [2] | Steele 2000 [3] |
| Developmental approach | Two-hundred forty-nine GW-deployed members of a Reserve construction battalion were surveyed in 4 cities of the southeastern U.S. where detachments were located, and a 2-stage principal components factor analysis of symptoms was performed. In the first stage, for each of the 27 typical GWI symptoms the Symptom Questionnaire included a battery of 4-20 clarifying questions (a total of 214 items). The items for a given symptom were analyzed by exploratory factor analysis to derive 2-3 orthogonal factor scales, generating a total of 52 unambiguous *symptom scales*. In the second stage, factor analysis with orthogonal rotation of the 52 unambiguous symptom scales generated 6 *syndrome scales*. | Members of 2 National Guard, 1 Reserve, and 1 active-duty units of the U.S. Air Force were surveyed by self-administered 35-question written questionnaires on military bases during training exercises. Exploratory factor analysis identified 2 factor scales in split halves of the database. Six symptom questions endorsed by ≥25% of the GW-deployed veterans and found to be 2.5 times more prevalent in the GW-deployed than the non-deployed and 4 more questions with high loadings on the factor analysis (10 questions in all) were intuitively grouped into 2 categories for *mood-cognitio*n and *musculoskeletal pain or stiffness*, and a third category for *fatigu*e was added because it was commonly found important by other studies. | Telephone interviews on 32 symptoms and 10 exclusionary conditions were conducted with 1,548 deployed and 482 non-deployed GW-era veterans living in Kansas. Each symptom was self-rated as mild, moderate or severe. For each of 6 symptom groups (respiratory, gastrointestinal, neuropsychological, sleep disturbances, pain and skin) a symptom group was considered positive if a veteran endorsed ≥2 of its symptoms of any severity or 1 symptom of at least moderate severity. Comparative analysis found that the proportion of veterans with any 1 or 2 positive symptom groups was similar in deployed and non-deployed samples, but for veterans reporting ≥3 positive symptom groups the proportion was significantly higher in the deployed sample. *A priori* 10 comorbid disease categories* that might have symptoms similar to GWI were designated as exclusionary conditions. |
| Final criteria | Each subject was assigned a value on each of the 6 syndrome factor scales by weighting all their questionnaire responses by the factor weights. The syndrome scales were dichotomized at 1.5 SD above the mean to form indicators of 6 GWI syndrome *variants*. The first 3 variants—1 (“impaired cognition”), 2 (“confusion-ataxia”), and 3 (“central pain”)—explained most of the variance (had high eigenvalues) and have been used in many clinical studies. Subjects meeting any of the 6 variants met the overall GWI *Research case definition* used in the present study. | A clinical diagnosis of chronic multisymptom illness is having ≥1 chronic symptom (present for ≥6 months) each from at least 2 of the following 3 symptom categories: 1) *fatigue*; 2) *mood and cognition* (symptoms of feeling depressed, difficulty remembering or concentrating, feeling moody, feeling anxious, trouble finding words, difficulty sleeping); and 3) *musculoskeletal* (symptoms of joint pain, joint stiffness, or muscle pain). | Veterans having 3 or more positive symptom groups, as defined above, satisfied the basic case definition. Those with any of the 10 exclusionary conditions* were reclassified to the non-GWI group to form the Original Steele case definition [3, 4]. As the GW veteran population aged and the exclusionary conditions became more common, concerns of bias introduced by the exclusion have been raised [5]. As a result, Modified Steele case definitions foregoing entirely or limiting the number of exclusionary conditions have been used [4, 6]. Consequently, in our study, we evaluate the original Steele definition (Modified Kansas with exclusions) and one without exclusions (Modified Kansas without exclusions). |
| Minimum number of symptoms required to identify a case | All 52 symptom scales developed from 214 GWI symptom questions contribute to the factor scales for each of the 6 syndrome variants. | 2 symptoms (1 symptom each from 2 of the 3 symptom categories) | 3 symptoms (1 symptom from each of 3 symptom groups) |
| Validation | Confirmatory factor analysis with a 3-factor structural equation model showed good fit by Hu-Bentler criteria [7] to the following 2 independent datasets: 1) 335 GW veterans identified in the GW clinic of a regional Veterans Affairs Hospital [8] and 2) both random halves of a population-representative sample of 6,497 deployed GW veterans [9]. | Procrustes factor analysis with oblique rotation validated the factor results. The 2 factor scales were dichotomized at the 75^th^ percentile to define “factor score cases,” which were similar to clinically diagnosed cases (*kappa*=0.79). No validation of the intuitively derived criteria used for the final CDC case definition was reported. | No validation studies were reported. |
| Methods of subclassifying subjects | The 3 GWI variants, defined by differences in symptom profiles identified by the PCA, were found to predict large differences in level of functional disability as well as epidemiologic risk factors, level of cognitive and autonomic dysfunction, and degree of abnormal brain function on neuroimaging and EEG tests. | The symptom survey questionnaire asked subjects to rate each symptom as mild, moderate or severe. A case was classified as CDC Severe if each of the case-defining symptoms was self-rated as severe; otherwise, the case was classified as mild-to-moderate in severity. This subclassification has rarely been used in studies using this case definition. | No subclassification was defined. |
| PCA, principal components analysis; EEG, electroencephalography. | | | |
| *Comorbid exclusionary conditions causing exclusion include cancer, diabetes, heart disease, chronic infectious disease, postwar injuries, liver disease, lupus, multiple sclerosis, stroke, or any serious psychiatric condition including post-traumatic stress disorder. | | | |

| **Table S2.** Comparison of symptom measures used by the 3 case definitions to define GWI | | | |
| --- | --- | --- | --- |
| **Research case definition** | | **Kansas case definition^c^** | **CDC case definition^d^** |
| **Raw symptoms (No. of descriptive questions)^a^** | **Derivative symptom scales^b^** |  |  |
| Chronic fatigue (9) | Excessive daytime sleepiness | Fatigue | Fatigue |
|  | Excessive muscle exhaustion | Exercise intolerance |  |
| Problems sleeping (7) | Early night insomnia | Problems sleeping | Difficulty sleeping |
|  | Middle/terminal insomnia | Unrefreshing sleep |  |
| Joint pains (12) | Hips and extremities | Joint pains | Joint pain |
|  | Neck and shoulders | Joint stiffness | Joint stiffness |
| Muscle pains (9) | Neck, shoulders, upper back | Muscle pain | Muscle pain |
|  | Lower back, buttocks, legs | Generalized body pain |  |
|  | Arms |  |  |
| Muscle weakness,  Bowel/bladder control (9) | Generalized weakness |  |  |
|  | Bladder control /leg weakness |  |  |
| Headaches (15) | Tension | Headaches |  |
|  | Migraine 1 |  |  |
|  | Migraine 2 |  |  |
| Dizzy spells,  Loss of balance or coordination (9) | Vertigo and ataxia | Dizziness |  |
|  | Incoordination of hands, arms |  |  |
|  | Difficulties with speaking | Word-finding difficulty | Trouble finding words |
| Tingling (15)  Numbness (15) | Face, tongue and lips | Tingling/numbness in extremities |  |
|  | Trunk and groin |  |  |
|  | Extremities |  |  |
| Fever, chills, hot flashes, night sweats (4) | Low-grade fever | Heat/cold intolerance |  |
|  | Night sweats | Night sweats |  |
| Symptoms triggered by smells (13) | Physical reactions | Symptoms from smells |  |
|  | Emotional reactions |  |  |
| Skin rash (44) | (Did not contribute to the PCA model) | Skin rashes |  |
|  |  | Other skin problems |  |
| Chronic diarrhea (6) | Frequent watery diarrhea | Diarrhea |  |
| Abdominal pain (6) | Abdominal pain, nausea | Nausea/stomach upset |  |
|  |  | Abdominal pain/cramps |  |
| Pulmonary symptoms (7) | Dyspnea and chest pain | Shortness of breath |  |
|  | Wheezing and coughing | Frequent coughing |  |
|  |  | Wheezing |  |
| Problems with thinking or reasoning (20) | Distractibility |  |  |
|  | Confusion |  |  |
|  | Mental processing |  |  |
|  | Disorientation |  |  |
| Attention or concentration (10) | Distractibility | Difficulty concentrating | Difficulty remembering or concentrating |
|  | Confusion |  |  |
| Memory problems (10) | Short-term memory | Short-term memory problems |  |
|  | Long-term memory |  |  |
|  | Can’t find parked car |  |  |
| Major change in emotional state (13) | Depression | Feeling down/depressed | Feeling depressed |
|  |  | Irritability/anger problem |  |
|  |  | Feeling moody | Feeling moody |
|  |  | Feeling anxious | Feeling anxious |
|  | Hallucinations |  |  |
| Problems with sexual functioning (10) | Impotence |  |  |
|  | Dyspareunia in wife |  |  |
| Swollen glands (9) | Neck and groin |  |  |
|  | Groin and around elbows |  |  |
|  | Around knees and elbows |  |  |
| Feelings triggered by being in tight places (13) | Physical symptoms |  |  |
|  | Emotional symptoms |  |  |
|  | Hyperventilation |  |  |
| A list of physician’s diagnoses (22) | Depression/anxiety disorders |  |  |
|  | Liver disease diagnosis |  |  |
|  | PTSD/depression diagnoses |  |  |
| Weight change (2) | Weight at survey minus weight at war |  |  |
| Tinnitus (0) | Tinnitus |  |  |
|  |  | Tremor |  |
|  |  | Sensitivity to light |  |
|  |  | Blurred/double vision |  |
| ^a^ Each of the 27 raw symptoms was obtained by a yes/no question followed by a battery of follow-up descriptive questions such as 15 anatomic locations of tingling or numbness. The responses to the follow-up descriptive questions were coded 0 for no, 1 for yes but no longer, and 2 for yes and continuing. | | | |
| ^b^ A separate first-stage principal components factor analysis of the follow-up descriptive questions for each raw symptom generated 1-4 orthogonal symptom scales that separated out distinct meanings of the ambiguous raw symptom. For example, “chronic fatigue” was parsed into 2 different types of fatigue, each represented by an ordinal factor scale: excessive daytime sleepiness and muscle exhaustion. A second-stage principal components factor analysis of these 52 ordinal symptom factor scales generated 6 syndrome factor scales that were dichotomized to form 6 syndrome variants (see **Figures S1 and S2**). Positive on any of the 6 variant indicators met the Research case definition for GWI. | | | |
| ^c^ Each of the 32 Kansas symptom questions required a yes/no answer followed by a self-rating of mild, moderate or severe. The questions were grouped into 6 domains, and GWI was defined by having ≥2 mild or ≥1 moderate or severe symptom in at least 3 of the 6 domains. | | | |
| ^d^ Each of the 10 CDC symptom questions required a yes/no answer followed by a self-rating of mild, moderate or severe. The questions were grouped into 3 domains, and GWI was defined by having ≥1 symptom in at least 2 of the 3 domains. | | | |


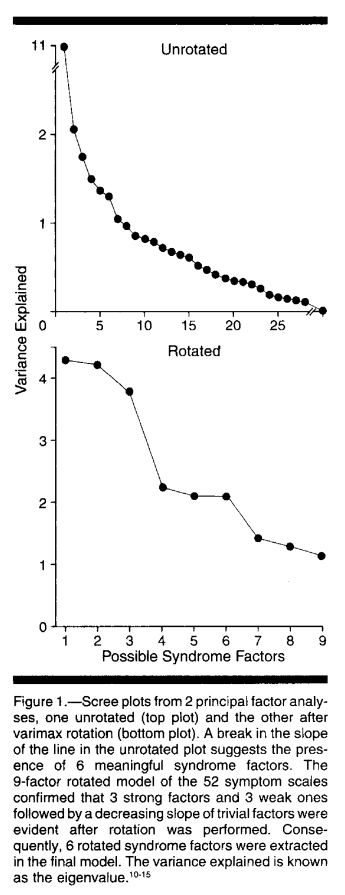


**Figure S1.** An unrotated scree plot (top) and a second one after varimax rotation (bottom) generated by the principal components factor analysis of the 52 symptom scales from the 249 Gulf War veterans in the Developmental Sample. A break in the slope of the line in the unrotated plot suggests the presence of 6 meaningful syndrome factors. The 9-factor rotated model of the 52 symptom scales confirmed 3 strong factors and 3 weak ones followed by a decreasing slope of trivial factors. Consequently, 6 rotated syndrome factors were extracted in the final model. The variance explained is known as the eigenvalue. The plots are reproduced from the original publication in JAMA [1] with permission of the publisher.


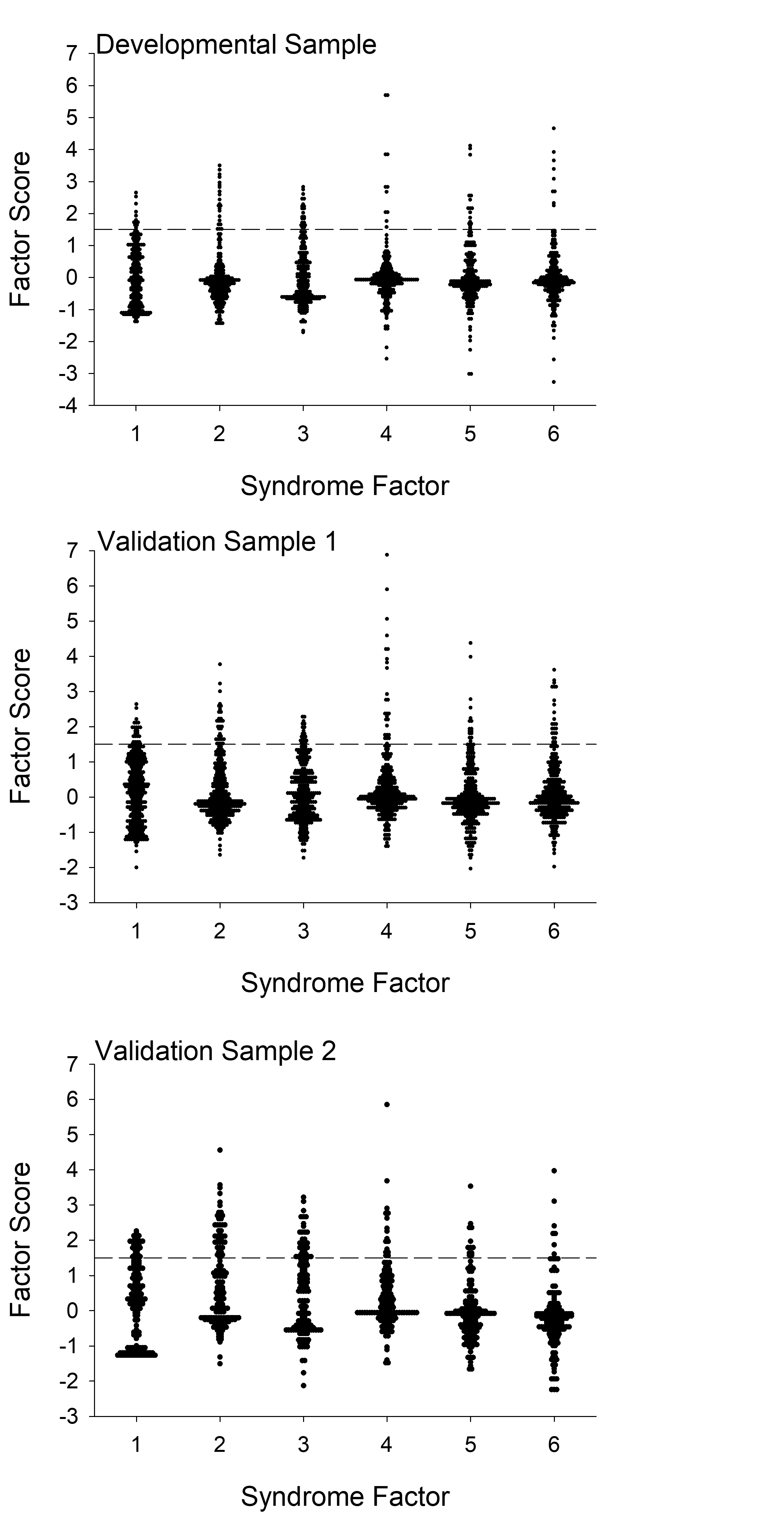


**Figure S2.** Distributions of the deployed Gulf War veterans on each of the 6 syndrome factor scales from the original Developmental study in the Naval Reserve Battalion [1] and the 2 validation samples [8, 9]. Each veteran’s value on a given syndrome factor scale was generated by summing the products of their response values on the 52 symptom scales and their factor weights from the principal components factor analysis. The Developmental Sample included 249 veterans, and the First Validation Sample included 335, but, since the 6,816 veterans in the Second Validation Sample could not be displayed in a dot density plot, the 158 randomly selected members of that sample studied in the clinical phases of the study were plotted here. The plot of the Developmental Sample is reproduced from the original publication in JAMA [1] with permission of the publisher.

| **Table S3.** Goodness-of-fit validation statistics for structural equation model of Gulf War illness with 3 first-order factors (syndrome variants) and a second-order factor (overall Gulf War illness),^a^ by study and sample within study | | | | | | |
| --- | --- | --- | --- | --- | --- | --- |
| Study and sample with study | | Sample size | Goodness-of-fit statistics | | | |
|  |  |  | SRMR^b^ | RMSEA^c^ | CFI^d^ | TJI^e^ |
| Criteria for a good fit[7] | |  | ≤0.080 | ≤0.060 | ≥0.950 | ≥0.950 |
| Deployed U.S. Reserve Naval Construction Battalion (developmental sample)[1] | | 249 | 0.043 | 0.023 | 0.992 | 0.988 |
| Deployed U.S. Army veterans (first validation sample)[8] | | 335 | 0.043 | 0.044 | 0.975 | 0.964 |
| U.S. Military Health Survey (second validation sample)^f^ | |  |  |  |  |  |
|  | Random half 1 | 3,408 | 0.054 | 0.018 | 0.968 | 0.954 |
|  | Random half 2 | 3,408 | 0.048 | 0.017 | 0.972 | 0.967 |
|  | Both halves combined | 6,816 | 0.048 | 0.017 | 0.970 | 0.958 |
|  | Forced equal loadings across both halves | 6,816 | 0.054 | 0.015 | 0.972 | 0.967 |
| ^a^Corresponds to model 3 developed in the first validation study.[8] | | | | | | |
| ^b^SRMR = Standardized root mean-square residual, an absolute fit index, analogous to R^2^ for a linear model, and the most sensitive to misspecification of factor covariances or latent structures; the remaining 3 fit indexes are most sensitive to misspecification of factor loadings [7]. | | | | | | |
| ^c^RMSEA = root mean-square error of approximation, an absolute fit index that adjusts fit by the number of model parameters estimated to prevent large complex model structures from inflating the fit [7]. Hu and Bentler reported that the combination of SRMR >0.09 and RMSEA >0.06 for rejection results in the least sum of type I and type II model rejection errors [7]. | | | | | | |
| ^d^CFI = comparative fit index, a type 3 incremental fit index that estimates the improvement in fit over a baseline null model where all measured variables are uncorrelated [7]. | | | | | | |
| ^e^TLI = Tucker-Lewis index (also Bentler-Bonett nonnormed fit index), a type 2 incremental fit index [7]. | | | | | | |
| ^f^All results are population estimates for the U.S. military population adjusted to correct for unequal selection probabilities from strata and minimize bias from nonlocation and nonparticipation by application of the survey analysis weights [9]. | | | | | | |
| This table is reproduced from *Neuroepidemiology*[9] with permission from the publisher, Copyright © 2013 Karger Publishers, Basel Switzerland. | | | | | | |

| **Table S4.** Demographic and military characteristics of the deployed population and the controls and GWI cases by the various definitions and their overlap | | | | | | | | | | |
| --- | --- | --- | --- | --- | --- | --- | --- | --- | --- | --- |
| **Population and alternative GWI case definitions^a^** | | **Estimated population prevalence rate (SE)^b^** | **Estimated population prevalence, N^b^** | **Prevalence case-control sample, N** | **Age, % >45** | **Sex, % female** | **Wartime service branch, % Army** | **Wartime rank, % enlisted** | **Active duty/Guard status, % active duty** | **Combat Exposure Scale, mean** |
| Total deployed force | | 100.0 (0.0) | 676,884 | 1698 | 45.7 | 16.1 | 53.2 | 90.6 | 66.1 | 1.19 |
| Unaffected controls | | 53.9 (1.4) | 364,727 | 508 | 45.0 | 6.1 | 35.8 | 84.3 | 63.0 | 1.09 |
| Research | | 13.6 (0.9) | 91,974 | 508 | 46.8 | 22.1 | 66.5 | 94.5 | 67.3 | 1.30 |
|  | Research variant 1 | 1.8 (0.2) | 11,947 | 94 | 45.4 | 30.9 | 68.1 | 91.5 | 62.8 | 1.31 |
|  | Research variant 2 | 6.1 (0.7) | 42,286 | 206 | 47.0 | 18.5 | 71.8 | 95.6 | 66.5 | 1.31 |
|  | Research variant 3 | 4.6 (0.8) | 31,031 | 163 | 47.9 | 19.6 | 67.5 | 93.9 | 71.2 | 1.29 |
| CDC | | 41.7 (1.3) | 282,398 | 1109 | 46.0 | 20.7 | 60.7 | 93.5 | 67.7 | 1.24 |
|  | CDC, not Research^c^ | 28.8 (1.2) | 195,180 | 624 | 45.3 | 19.7 | 56.1 | 92.8 | 67.8 | 1.18 |
| CDC mild-to-moderate | | 31.1 (1.3) | 210,775 | 757 | 45.8 | 20.9 | 57.6 | 93.0 | 66.7 | 1.17 |
|  | CDC mild-to-moderate, not Research^c^ | 24.5 (1.1) | 166,044 | 521 | 45.5 | 18.6 | 54.9 | 92.3 | 66.4 | 1.16 |
| CDC Severe | | 10.6 (1.0) | 71,623 | 352 | 46.5 | 20.2 | 67.3 | 94.6 | 69.9 | 1.39 |
|  | CDC Severe, not Research^c^ | 4.3 (0.7) | 29,136 | 103 | 44.6 | 25.2 | 62.1 | 95.2 | 74.8 | 1.30 |
| Modified Kansas without exclusions | | 39.0 (1.3) | 263,761 | 1090 | 45.9 | 21.0 | 61.7 | 94.0 | 67.8 | 1.23 |
|  | Modified Kansas without exclusions, not Research^c^ | 26.0 (1.1) | 176,084 | 602 | 45.2 | 20.1 | 58.0 | 93.7 | 68.4 | 1.18 |
| Modified Kansas with exclusions | | 25.6 (1.2) | 173,129 | 748 | 44.8 | 22.2 | 60.2 | 93.7 | 70.2 | 1.20 |
|  | Modified Kansas with exclusions, not Research^c^ | 19.3 (1.0) | 130,675 | 509 | 44.6 | 20.6 | 58.2 | 92.9 | 69.3 | 1.19 |
| ^a^ Figure 1 illustrates the overlap of the case definitions. | | | | | | | | | | |
| ^b^ The parameters and standard errors of the deployed population were estimated from the stratified random population sample of the USMHS allowing for the complex sample design. | | | | | | | | | | |
| ^c^ These groups contain those military personnel meeting the CDC or Kansas case definitions but excluding those meeting the Research definition. | | | | | | | | | | |


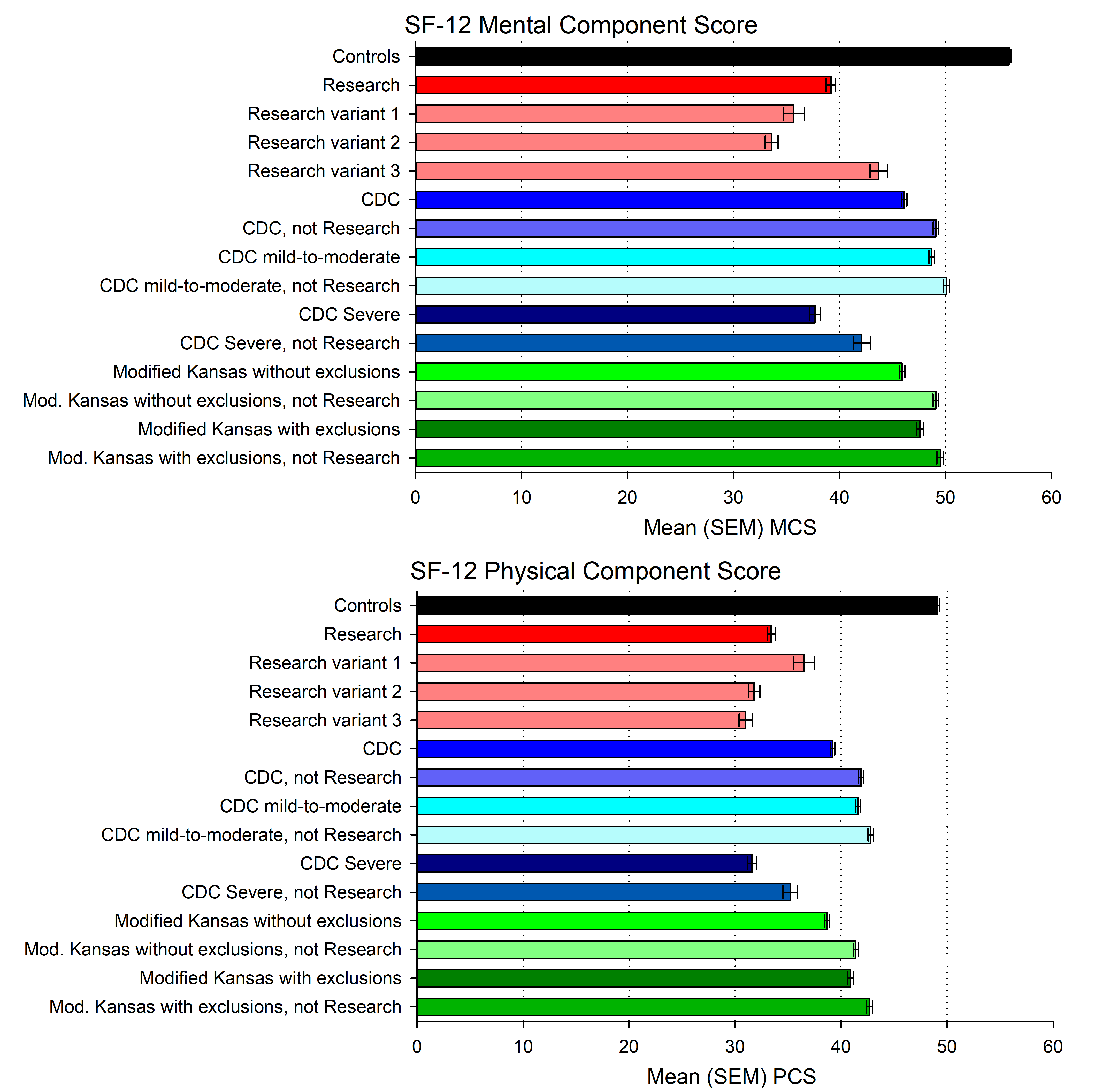


**Figure S3.** Mean (SEM) SF-12 Mental and Physical Component Scores by GWI case definition in 6,497 deployed veterans. Lower scores indicate worse health-related quality of life and impaired functionality

| **Table S5.** Mean SF-12 Mental and Physical Component Scores by GWI case definitions measured in 6,497 deployed Gulf War veterans | | | | | | | |
| --- | --- | --- | --- | --- | --- | --- | --- |
| **Population and alternative GWI case definitions^a^** | | **Sample size** | **Mental Component Score** | |  | **Physical Component Score** | |
|  |  |  | **Mean** | **SEM** |  | **Mean** | **SEM** |
| Unaffected controls | | 1,952 | 56.0 | 0.18 |  | 49.1 | 0.19 |
| Research | | 834 | 39.2 | 0.45 |  | 33.4 | 0.38 |
|  | Research variant 1 | 133 | 35.7 | 1.00 |  | 36.5 | 0.99 |
|  | Research variant 2 | 342 | 33.6 | 0.61 |  | 31.8 | 0.56 |
|  | Research variant 3 | 262 | 43.7 | 0.82 |  | 31.0 | 0.62 |
| CDC | | 2,649 | 46.1 | 0.25 |  | 39.2 | 0.22 |
|  | CDC, not Research^a^ | 1,853 | 49.1 | 0.27 |  | 41.9 | 0.24 |
| CDC mild-to-moderate | | 2,019 | 48.7 | 0.26 |  | 41.6 | 0.23 |
|  | CDC mild or moderate, not Research^a^ | 1,630 | 50.1 | 0.27 |  | 42.8 | 0.25 |
| CDC Severe | | 630 | 37.7 | 0.51 |  | 31.6 | 0.40 |
|  | CDC Severe, not Research^a^ | 223 | 42.1 | 0.81 |  | 35.2 | 0.68 |
| Modified Kansas without exclusions | | 2,493 | 45.9 | 0.26 |  | 38.7 | 0.22 |
|  | Kansas without exclusions, not Research^a^ | 1,694 | 49.1 | 0.28 |  | 41.4 | 0.25 |
| Modified Kansas with exclusions | | 1,642 | 47.6 | 0.30 |  | 40.9 | 0.26 |
|  | Kansas with exclusions, not Research^a^ | 1,261 | 49.5 | 0.32 |  | 42.7 | 0.28 |
| ^a^ These groups contain veterans meeting the various CDC or Kansas definitions but not the Research definition. | | | | | | | |

| **Table S6.**  The association of having heard nerve agent alarms in the Gulf War with the various case definitions of Gulf War illness and their overlap, estimated by unweighted logistic regression in the full deployed USMHS sample, adjusted for the confounding variables age, sex, service branch, rank, active duty vs Guard/Reserve, special strata, and combat exposure scale (numerical values for Figure 3) | | | |
| --- | --- | --- | --- |
| Alternative case definitions | | No. of GWI cases in the USMHS deployed sample^a^ | Adjusted^b^ odds ratio (95% CI) |
| Research | | 830 | 4.14 (3.41-5.02) |
|  | Research variant 1 | 134 | 4.08 (2.65-9.35) |
|  | Research variant 2 | 343 | 6.74 (4.86-9.20) |
|  | Research variant 3 | 257 | 3.38 (2.49-4.57) |
| CDC | | 2,631 | 2.38 (2.13-2.67) |
|  | CDC, not Research^c^ | 1,840 | 1.96 (1.73-2.22) |
| CDC mild or moderate | | 2,006 | 2.12 (1.88-2.40) |
|  | CDC mild or moderate, not Research^c^ | 1,618 | 1.94 (1.70-2.20) |
| CDC Severe | | 625 | 3.89 (3.14-4.83) |
|  | CDC Severe, not Research^c^ | 222 | 2.14 (1.59-2.89) |
| Kansas without exclusions | | 2,480 | 2.55 (2.27-2.87) |
|  | Kansas without exclusions, not Research^c^ | 1,684 | 2.14 (1.88-2.43) |
| Kansas with exclusions | | 1,625 | 2.35 (2.06-2.68) |
|  | Kansas with exclusions, not Research^c^ | 1,248 | 2.12 (1.84-2.45) |
| ^a^ There were 3,437 controls not meeting any of the GWI case definitions. | | | |
| ^b^Adjusted for age, sex, service branch, rank, active duty vs Guard/Reserve, special strata, and combat exposure scale | | | |
| ^c^ These groups contain veterans meeting the various CDC or Kansas definitions but not the Research definition. | | | |

| **Table S7.** Percentage distribution of the *PON1* Q192R genotype in the unaffected controls and groups of cases defined by the alternative GWI case definitions in the genetics subsample of the USMHS (numerical values for Figure 4) | | | | | |
| --- | --- | --- | --- | --- | --- |
|  | | No. of deployed veterans in the genetics subsample^a^ | Percentage distribution of subjects by *PON1* Q192R genotype | | |
| Alternative case definitions | |  | QQ | QR | RR |
| Unaffected controls | | 508 | 46.1 | 42.5 | 11.4 |
| Research | | 508 | 33.9 | 44.7 | 21.5 |
|  | Research variant 1 | 94 | 41.5 | 41.5 | 17.0 |
|  | Research variant 2 | 206 | 33.5 | 45.6 | 20.9 |
|  | Research variant 3 | 163 | 29.5 | 48.5 | 22.1 |
| CDC | | 1109 | 37.1 | 44.5 | 18.4 |
|  | CDC, not Research^b^ | 624 | 38.9 | 44.9 | 16.2 |
| CDC mild or moderate | | 757 | 37.5 | 45.1 | 17.4 |
|  | CDC mild or moderate, not Research^b^ | 521 | 38.2 | 45.7 | 16.2 |
| CDC Severe | | 352 | 36.1 | 43.5 | 20.5 |
|  | CDC Severe, not Research^b^ | 103 | 42.7 | 40.8 | 16.5 |
| Kansas without exclusions | | 1090 | 36.2 | 45.1 | 18.9 |
|  | Kansas without exclusions, not Research^b^ | 602 | 37.5 | 45.5 | 16.9 |
| Kansas with exclusions | | 748 | 36.8 | 44.8 | 18.5 |
|  | Kansas with exclusions, not Research^b^ | 509 | 37.5 | 45.2 | 17.3 |
| ^a^The genetics analyses were performed on a prevalence case-control subsample of all deployed participants in the USMHS sample who met the GWI Research or Kansas case definitions and a random subsample of those meeting the CDC case definition and all others not meeting any of the case definitions and who consented to provide a blood sample. | | | | | |
| ^b^ These groups contain veterans meeting the various CDC or Kansas definitions but not the Research definition. | | | | | |

| **Table S8.** Interaction on the additive and multiplicative scales of hearing nerve agent alarms and *PON1* Q192R genotype on GWI by the **original Research** case definition | | | | | | | | | |
| --- | --- | --- | --- | --- | --- | --- | --- | --- | --- |
| Heard nerve agent alarms | | *PON1* Q192R genotype | | | | | |  | |
|  |  | QQ | | QR | | RR | | PORs for *PON1* Q192R genotypes within strata of alarms | |
|  |  | N  cases/ controls | POR (95%CI) | N  cases/ controls | POR (95%CI) | N  cases/ controls | POR (95%CI) | QR vs QQ | RR vs QQ |
|  | No | 43/130 | 1.0 | 50/120 | 1.26 (0.78-2.03) | 18/37 | 1.47 (0.76-2.85) | 1.26 (0.78-2.03) | 1.47 (0.76-2.85) |
|  | Yes | 129/104 | 3.75 (2.44-5.77) | 177/96 | 5.57 (3.64-8.53) | 91/21 | 13.10 (7.29-23.55) | 1.49 (1.04-2.13) | 3.49 (2.04-6.00) |
| POR (95%CI) for alarms within strata of genotypes | |  | 3.75 (2.44-5.77) |  | 4.43 (2.93-6.69) |  | 8.91 (4.27-18.60) |  |  |
| Additive scale: RERI (95%CI)  Unadjusted  Adjusted for confounders | | | 1.0  1.0 | 1.56 (-0.28 – 3.73)  1.95 (-0.08 – 4.86) | | 8.88 (3.77 – 18.61)  7.69 (2.71 – 19.13) | |  |  |
| Multiplicative scale: POR (95%CI) from LR interaction term  Unadjusted  Adjusted for confounders | | | 1.0  1.0 | 1.18 (0.65 – 2.14)  1.45 (0.70 – 2.97) | | 2.38 (1.01 – 5.57)  3.41 (1.20 – 9.72) | |  |  |
| Note: The RERI is a measure of interaction on the additive scale; it has a distribution of minus infinity to plus infinity with 0 as the equivalency point indicating no additive interaction. The prevalence odds ratio (POR) has a distribution of zero to plus infinity with 1 as the equivalency point. The potential confounders controlled for in the adjusted models include: age, sex, service branch, rank, active duty vs Guard/Reserve, special strata, and combat exposure scale. The analyses included 508 cases and 508 controls. Abbreviations: aRERI, relative excess risk due to interaction adjusted for measured confounding; CI, confidence interval; LR, logistic regression; PON1, paraoxonase-1. | | | | | | | | | |

| **Table S9.** Interaction on the additive and multiplicative scales of hearing nerve agent alarms and *PON1* Q192R genotype on GWI by the **Research Variant 1** case definition | | | | | | | | | |
| --- | --- | --- | --- | --- | --- | --- | --- | --- | --- |
| Heard nerve agent alarms | | *PON1* Q192R genotype | | | | | |  | |
|  |  | QQ | | QR | | RR | | PORs for *PON1* Q192R genotypes within strata of alarms | |
|  |  | N  cases/ controls | POR (95%CI) | N  cases/ controls | POR (95%CI) | N  cases/ controls | POR (95%CI) | QR vs QQ | RR vs QQ |
|  | No | 11/130 | 1.0 | 6/120 | 0.59 (0.21-1.65) | 3/37 | 0.96 (0.25-3.62) | 0.59 (0.21-1.65) | 0.96 (0.25-3.62) |
|  | Yes | 28/104 | 3.18 (1.51-6.69) | 33/96 | 4.06 (1.95-9.44) | 13/21 | 7.32 (2.90-18.47) | 1.28 (0.72-2.27) | 2.30 (1.03-5.16) |
| POR (95%CI) for alarms within strata of genotypes | |  | 3.18 (1.51-6.69) |  | 6.875 (2.77-17.01) |  | 7.64 (1.95-29.89) |  |  |
| Additive scale: RERI (95%CI)  Unadjusted  Adjusted for confounders | | | 1.0  1.0 | 1.29 (-1.71 – 4.59)  1.78 (-0.67 – 5.49) | | 4.18 (-0.49 – 14.33)  3.66 (0.17 – 13.66) | |  |  |
| Multiplicative scale: POR (95%CI) from LR interaction term  Unadjusted  Adjusted for confounders | | | 1.0  1.0 | 2.16 (0.67 – 7.00)  3.25 (0.88 – 12.00) | | 2.40 (0.51 – 11.40)  5.03 (0.84 – 30.20) | |  |  |
| Note: The RERI is a measure of interaction on the additive scale; it has a distribution of minus infinity to plus infinity with 0 as the equivalency point indicating no additive interaction. The prevalence odds ratio (POR) has a distribution of zero to plus infinity with 1 as the equivalency point. The potential confounders controlled for in the adjusted models include: age, sex, service branch, rank, active duty vs Guard/Reserve, special strata, and combat exposure scale. The analyses included 94 cases and 508 controls. Abbreviations: aRERI, relative excess risk due to interaction adjusted for measured confounding; CI, confidence interval; LR, logistic regression; PON1, paraoxonase-1. | | | | | | | | | |
|  | | | | | | | | | |

| **Table S10.** Interaction on the additive and multiplicative scales of hearing nerve agent alarms and *PON1* Q192R genotype on GWI by the **Research Variant 2** case definition | | | | | | | | | |
| --- | --- | --- | --- | --- | --- | --- | --- | --- | --- |
| Heard nerve agent alarms | | *PON1* Q192R genotype | | | | | |  | |
|  |  | QQ | | QR | | RR | | PORs for *PON1* Q192R genotypes within strata of alarms | |
|  |  | N  cases/ controls | POR (95%CI) | N  cases/ controls | POR (95%CI) | N  cases/ controls | POR (95%CI) | QR vs QQ | RR vs QQ |
|  | No | 10/130 | 1.0 | 15/120 | 1.63 (0.70-3.76) | 4/37 | 1.41 (0.42-4.74) | 1.63 (0.70-3.76) | 1.41 (0.42-4.74) |
|  | Yes | 59/104 | 7.38 (3.60-15.12) | 79/96 | 10.70 (5.27-21.73) | 39/21 | 24.14 (10.49-55.57) | 1.45 (0.94-2.25) | 3.27 (1.76-6.08) |
| POR (95%CI) for alarms within strata of genotypes | |  | 7.38 (3.60-15.12) |  | 6.58 (3.56-12.16) |  | 17.18 5.39-54.80) |  |  |
| Additive scale: RERI (95%CI)  Unadjusted  Adjusted for confounders | | | 1.0  1.0 | 2.70 (-2.98 – 10.43)  3.60 (-2.71 – 14.62) | | 16.36 (6.43 – 44.95)  15.84 (5.36 – 56.31) | |  |  |
| Multiplicative scale: POR (95%CI) from LR interaction term  Unadjusted  Adjusted for confounders | | | 1.0  1.0 | 0.89 (0.35 – 2.30)  1.09 (0.37 – 3.25) | | 2.33 (0.60 – 9.11)  3.78 (0.75 – 19.00) | |  |  |
| Note: The RERI is a measure of interaction on the additive scale; it has a distribution of minus infinity to plus infinity with 0 as the equivalency point indicating no additive interaction. The prevalence odds ratio (POR) has a distribution of zero to plus infinity with 1 as the equivalency point. The potential confounders controlled for in the adjusted models include: age, sex, service branch, rank, active duty vs Guard/Reserve, special strata, and combat exposure scale. The analyses included 206 cases and 508 controls. Abbreviations: aRERI, relative excess risk due to interaction adjusted for measured confounding; CI, confidence interval; LR, logistic regression; PON1, paraoxonase-1. | | | | | | | | | |
|  | | | | | | | | | |

| **Table S11.** Interaction on the additive and multiplicative scales of hearing nerve agent alarms and *PON1* Q192R genotype on GWI by the **Research Variant 3** case definition | | | | | | | | | |
| --- | --- | --- | --- | --- | --- | --- | --- | --- | --- |
| Heard nerve agent alarms | | *PON1* Q192R genotype | | | | | |  | |
|  |  | QQ | | QR | | RR | | PORs for *PON1* Q192R genotypes within strata of alarms | |
|  |  | N  cases/ controls | POR (95%CI) | N  cases/ controls | POR (95%CI) | N  cases/ controls | POR (95%CI) | QR vs QQ | RR vs QQ |
|  | No | 16/130 | 1.0 | 20/120 | 1.35 (0.67-2.73) | 7/37 | 1.54 (0.59-4.02) | 1.35 (0.67-2.73) | 1.54 (0.59-4.02) |
|  | Yes | 32/104 | 2.50 (1.30-4.80) | 59/96 | 4.99 (2.71-9.21) | 29/21 | 11.22 (5.22-24.11) | 2.00 (1.20-3.33) | 4.49 (2.264-8.92) |
| POR (95%CI) for alarms within strata of genotypes | |  | 2.50 (1.30-4.80) |  | 3.69 (2.08-6.55) |  | 7.30 (2.73-19.52) |  |  |
| Additive scale: RERI (95%CI)  Unadjusted  Adjusted for confounders | | | 1.0  1.0 | 2.14 (-0.12 – 5.34)  2.77 (0.53 – 7.18) | | 8.18 (3.03 – 20.17)  7.93 (2.38 – 24.16) | |  |  |
| Multiplicative scale: POR (95%CI) from LR interaction term  Unadjusted  Adjusted for confounders | | | 1.0  1.0 | 1.48 (0.62 – 3.52)  2.42 (0.85 – 6.88) | | 2.92 (0.90 – 9.51)  4.54 (0.97 – 19.40) | |  |  |
| Note: The RERI is a measure of interaction on the additive scale; it has a distribution of minus infinity to plus infinity with 0 as the equivalency point indicating no additive interaction. The prevalence odds ratio (POR) has a distribution of zero to plus infinity with 1 as the equivalency point. The potential confounders controlled for in the adjusted models include: age, sex, service branch, rank, active duty vs Guard/Reserve, special strata, and combat exposure scale. The analyses included 163 cases and 508 controls. Abbreviations: aRERI, relative excess risk due to interaction adjusted for measured confounding; CI, confidence interval; LR, logistic regression; PON1, paraoxonase-1. | | | | | | | | | |
|  | | | | | | | | | |

| **Table S12.** Interaction on the additive and multiplicative scales of hearing nerve agent alarms and *PON1* Q192R genotype on GWI by the **CDC** case definition | | | | | | | | | |
| --- | --- | --- | --- | --- | --- | --- | --- | --- | --- |
| Heard nerve agent alarms | | *PON1* Q192R genotype | | | | | |  | |
|  |  | QQ | | QR | | RR | | PORs for *PON1* Q192R genotypes within strata of alarms | |
|  |  | N  cases/ controls | POR (95%CI) | N  cases/ controls | POR (95%CI) | N  cases/ controls | POR (95%CI) | QR vs QQ | RR vs QQ |
|  | No | 131/130 | 1.0 | 150/120 | 1.24 (0.88-1.75) | 50/37 | 1.34 (0.82-2.19) | 1.24 (0.88-1.75) | 1.34 (0.82-2.19) |
|  | Yes | 280/104 | 2.67 (1.92-3.72) | 344/96 | 3.56 (2.55-4.96) | 154/21 | 7.28 (4.34-12.20) | 1.33 (0.97-1.83) | 2.72 (1.64-4.53) |
| POR (95%CI) for alarms within strata of genotypes | |  | 2.67 (1.92-3.72) |  | 2.87 (2.06-3.99) |  | 5.43 (2.91-10.12) |  |  |
| Additive scale: RERI (95%CI)  Unadjusted  Adjusted for confounders | | | 1.0  1.0 | 0.64 (-0.48 – 1.78)  0.79 (-0.21 – 1.85) | | 4.26 (1.47 – 8.90)  2.92 (0.96 – 6.38) | |  |  |
| Multiplicative scale: POR (95%CI) from LR interaction term  Unadjusted  Adjusted for confounders | | | 1.0  1.0 | 1.07 (0.67 – 1.71)  1.34 (0.79 – 2.27) | | 2.03 (1.00 – 4.11)  2.95 (1.33 – 6.55) | |  |  |
| Note: The RERI is a measure of interaction on the additive scale; it has a distribution of minus infinity to plus infinity with 0 as the equivalency point indicating no additive interaction. The prevalence odds ratio (POR) has a distribution of zero to plus infinity with 1 as the equivalency point. The potential confounders controlled for in the adjusted models include: age, sex, service branch, rank, active duty vs Guard/Reserve, special strata, and combat exposure scale. The analyses included 1109 cases and 508 controls. Abbreviations: aRERI, relative excess risk due to interaction adjusted for measured confounding; CI, confidence interval; LR, logistic regression; PON1, paraoxonase-1. | | | | | | | | | |
|  | | | | | | | | | |

| **Table S13.** Interaction on the additive and multiplicative scales of hearing nerve agent alarms and *PON1* Q192R genotype on GWI by the **CDC** **mild-to-moderate** case definition | | | | | | | | | |
| --- | --- | --- | --- | --- | --- | --- | --- | --- | --- |
| Heard nerve agent alarms | | *PON1* Q192R genotype | | | | | |  | |
|  |  | QQ | | QR | | RR | | PORs for *PON1* Q192R genotypes within strata of alarms | |
|  |  | N  cases/ controls | POR (95%CI) | N  cases/ controls | POR (95%CI) | N  cases/ controls | POR (95%CI) | QR vs QQ | RR vs QQ |
|  | No | 102/130 | 1.0 | 117/120 | 1.24 (0.86-1.79) | 37/37 | 1.28 (0.75-2.15) | 1.24 (0.86-1.79) | 1.28 (0.75-2.15) |
|  | Yes | 182/104 | 2.23 (1.57-3.18) | 224/96 | 2.97 (2.09-4.23) | 95/21 | 5.77 (3.36-9.88) | 1.33 (0.95-1.87) | 2.59 (1.52-4.39) |
| POR (95%CI) for alarms within strata of genotypes | |  | 2.23 (1.57-3.18) |  | 2.39 (1.69-3.39) |  | 4.52 (2.35-8.72) |  |  |
| Additive scale: RERI (95%CI)  Unadjusted  Adjusted for confounders | | | 1.0  1.0 | 0.50 (-0.55 – 1.51)  0.64 (-0.33 – 1.60) | | 3.26 (0.93 – 7.13)  2.41 (0.68 – 5.46) | |  |  |
| Multiplicative scale: POR (95%CI) from LR interaction term  Unadjusted  Adjusted for confounders | | | 1.0  1.0 | 1.34 (0.74 – 2.44)  1.31 (0.75 – 2.27) | | 2.03 (0.96 – 4.28)  2.90 (1.25 – 6.71) | |  |  |
| Note: The RERI is a measure of interaction on the additive scale; it has a distribution of minus infinity to plus infinity with 0 as the equivalency point indicating no additive interaction. The prevalence odds ratio (POR) has a distribution of zero to plus infinity with 1 as the equivalency point. The potential confounders controlled for in the adjusted models include: age, sex, service branch, rank, active duty vs Guard/Reserve, special strata, and combat exposure scale. The analyses included 757 cases and 508 controls. Abbreviations: aRERI, relative excess risk due to interaction adjusted for measured confounding; CI, confidence interval; LR, logistic regression; PON1, paraoxonase-1. | | | | | | | | | |
|  | | | | | | | | | |

| **Table S14.** Interaction on the additive and multiplicative scales of hearing nerve agent alarms and *PON1* Q192R genotype on GWI by the **CDC Severe** case definition | | | | | | | | | |
| --- | --- | --- | --- | --- | --- | --- | --- | --- | --- |
| Heard nerve agent alarms | | *PON1* Q192R genotype | | | | | |  | |
|  |  | QQ | | QR | | RR | | PORs for *PON1* Q192R genotypes within strata of alarms | |
|  |  | N  cases/ controls | POR (95%CI) | N  cases/ controls | POR (95%CI) | N  cases/ controls | POR (95%CI) | QR vs QQ | RR vs QQ |
|  | No | 29/130 | 1.0 | 33/120 | 1.23 (0.71-2.15) | 13/37 | 1.58 (0.75-3.33) | 1.23 (0.71-2.15) | 1.58 (0.75-3.33) |
|  | Yes | 98/104 | 4.22 (2.59-6.88) | 120/96 | 5.60 (3.45-9.09) | 59/21 | 12.59 (6.64-23.89) | 1.33 (0.90-1.95) | 2.98 (1.69-5.27) |
| POR (95%CI) for alarms within strata of genotypes | |  | 4.22 (2.59-6.88) |  | 4.55 (2.84-7.27) |  | 8.00 (3.58-17.88) |  |  |
| Additive scale: RERI (95%CI)  Unadjusted  Adjusted for confounders | | | 1.0  1.0 | 1.15 (-1.13 – 3.62)  1.72 (-0.54 – 4.90) | | 7.80 (2.67 – 18.13)  7.16 (2.34 – 19.18) | |  |  |
| Multiplicative scale: POR (95%CI) from LR interaction term  Unadjusted  Adjusted for confounders | | | 1.0  1.0 | 1.08 (0.55 – 2.12)  1.55 (0.68 – 3.49) | | 1.89 (0.74 – 4.85)  3.61 (1.13 – 11.60) | |  |  |
| Note: The RERI is a measure of interaction on the additive scale; it has a distribution of minus infinity to plus infinity with 0 as the equivalency point indicating no additive interaction. The prevalence odds ratio (POR) has a distribution of zero to plus infinity with 1 as the equivalency point. The potential confounders controlled for in the adjusted models include: age, sex, service branch, rank, active duty vs Guard/Reserve, special strata, and combat exposure scale. The analyses included 352 cases and 508 controls. Abbreviations: aRERI, relative excess risk due to interaction adjusted for measured confounding; CI, confidence interval; LR, logistic regression; PON1, paraoxonase-1. | | | | | | | | | |
|  | | | | | | | | | |

| **Table S15.** Interaction on the additive and multiplicative scales of hearing nerve agent alarms and *PON1* Q192R genotype on GWI by the **Modified Kansas without** **exclusions** case definition | | | | | | | | | |
| --- | --- | --- | --- | --- | --- | --- | --- | --- | --- |
| Heard nerve agent alarms | | *PON1* Q192R genotype | | | | | |  | |
|  |  | QQ | | QR | | RR | | PORs for *PON1* Q192R genotypes within strata of alarms | |
|  |  | N  cases/ controls | POR (95%CI) | N  cases/ controls | POR (95%CI) | N  cases/ controls | POR (95%CI) | QR vs QQ | RR vs QQ |
|  | No | 116/130 | 1.0 | 153/120 | 1.43 (1.01-2.02) | 50/37 | 1.51 (0.93-2.48) | 1.43 (1.01-2.02) | 1.51 (0.93-2.48) |
|  | Yes | 278/104 | 3.00 (2.14-4.20) | 338/96 | 3.95 (2.82-5.53) | 155/21 | 8.27 (4.92-13.91) | 1.32 (0.96-1.81) | 2.76 (1.66-4.59) |
| POR (95%CI) for alarms within strata of genotypes | |  | 3.00 (2.14-4.20) |  | 2.76 (1.99-3.84) |  | 5.46 (2.93-10.19) |  |  |
| Additive scale: RERI (95%CI)  Unadjusted  Adjusted for confounders | | | 1.0  1.0 | 0.52 (-0.75 – 1.79)  0.58 (-0.61 – 1.79) | | 4.76 (1.59 – 10.06)  3.84 (1.30 – 8.52) | |  |  |
| Multiplicative scale: POR (95%CI) from LR interaction term  Unadjusted  Adjusted for confounders | | | 1.0  1.0 | 0.92 (0.58 – 1.48)  1.07 (0.62 – 1.85) | | 1.82 (0.90 – 5.57)  2.70 (1.20 – 6.08) | |  |  |
| Note: The RERI is a measure of interaction on the additive scale; it has a distribution of minus infinity to plus infinity with 0 as the equivalency point indicating no additive interaction. The prevalence odds ratio (POR) has a distribution of zero to plus infinity with 1 as the equivalency point. The potential confounders controlled for in the adjusted models include: age, sex, service branch, rank, active duty vs Guard/Reserve, special strata, and combat exposure scale. The analyses included 1090 cases and 508 controls. Abbreviations: aRERI, relative excess risk due to interaction adjusted for measured confounding; CI, confidence interval; LR, logistic regression; PON1, paraoxonase-1. | | | | | | | | | |
|  | | | | | | | | | |

| **Table S16.** Interaction on the additive and multiplicative scales of hearing nerve agent alarms and *PON1* Q192R genotype on GWI by the **Modified Kansas with exclusions** case definition | | | | | | | | | |
| --- | --- | --- | --- | --- | --- | --- | --- | --- | --- |
| Heard nerve agent alarms | | *PON1* Q192R genotype | | | | | |  | |
|  |  | QQ | | QR | | RR | | PORs for *PON1* Q192R genotypes within strata of alarms | |
|  |  | N  cases/ controls | POR (95%CI) | N  cases/ controls | POR (95%CI) | N  cases/ controls | POR (95%CI) | QR vs QQ | RR vs QQ |
|  | No | 91/130 | 1.0 | 113/120 | 1.35 (0.93-1.95) | 36/37 | 1.39 (0.82-2.37) | 1.35 (0.93-1.95) | 1.39 (0.82-2.37) |
|  | Yes | 184/104 | 2.53 (1.76-3.62) | 222/96 | 3.30 (2.31-4.73) | 102/21 | 6.94 (4.04-11.91) | 1.31 (0.93-1.84) | 2.75 (1.62-4.65) |
| POR (95%CI) for alarms within strata of genotypes | |  | 2.53 (1.76-3.62) |  | 2.46 (1.73-3.49) |  | 4.99 (2.59-9.63) |  |  |
| Additive scale: RERI (95%CI)  Unadjusted  Adjusted for confounders | | | 1.0  1.0 | 0.43 (-0.76 – 1.56)  0.56 (-0.52 – 1.60) | | 4.02 (1.26 – 8.69)  3.42 (1.20 – 7.56) | |  |  |
| Multiplicative scale: POR (95%CI) from LR interaction term  Unadjusted  Adjusted for confounders | | | 1.0  1.0 | 0.97 (0.59 – 1.61)  1.21 (0.68 – 2.16) | | 1.97 (0.99 – 4.18)  3.18 (1.35 – 7.49) | |  |  |
| Note: The RERI is a measure of interaction on the additive scale; it has a distribution of minus infinity to plus infinity with 0 as the equivalency point indicating no additive interaction. The prevalence odds ratio (POR) has a distribution of zero to plus infinity with 1 as the equivalency point. The potential confounders controlled for in the adjusted models include: age, sex, service branch, rank, active duty vs Guard/Reserve, special strata, and combat exposure scale. The analyses included 748 cases and 508 controls. Abbreviations: aRERI, relative excess risk due to interaction adjusted for measured confounding; CI, confidence interval; LR, logistic regression; PON1, paraoxonase-1. | | | | | | | | | |
|  | | | | | | | | | |

| **Table S17.** Interaction on the additive and multiplicative scales of hearing nerve agent alarms and *PON1* Q192R genotype on GWI including cases meeting the **CDC case definition excluding those meeting the Research** case definition | | | | | | | | | |
| --- | --- | --- | --- | --- | --- | --- | --- | --- | --- |
| Heard nerve agent alarms | | *PON1* Q192R genotype | | | | | |  | |
|  |  | QQ | | QR | | RR | | PORs for *PON1* Q192R genotypes within strata of alarms | |
|  |  | N  cases/ controls | POR (95%CI) | N  cases/ controls | POR (95%CI) | N  cases/ controls | POR (95%CI) | QR vs QQ | RR vs QQ |
|  | No | 90/130 | 1.0 | 106/120 | 1.28 (0.88-1.86) | 33/37 | 1.29 (0.75-2.21) | 1.28 (0.88-1.86) | 1.29 (0.75-2.21) |
|  | Yes | 153/104 | 2.13 (1.47-3.07) | 174/96 | 2.62 (1.82-3.78) | 68/21 | 4.68 (2.68-8.17) | 1.23 (0.87-1.75) | 2.20 (1.27-3.81) |
| POR (95%CI) for alarms within strata of genotypes | |  | 2.13 (1.47-3.07) |  | 2.05 (1.43-2.94) |  | 3.63 (1.84-7.15) |  |  |
| Additive scale: RERI (95%CI)  Unadjusted  Adjusted for confounders | | | 1.0  1.0 | 0.21 (-0.84 – 1.17)  0.51 (-0.44 – 1.40) | | 2.26 (0.22 – 5.53)  1.98 (0.42 – 4.67) | |  |  |
| Multiplicative scale: POR (95%CI) from LR interaction term  Unadjusted  Adjusted for confounders | | | 1.0  1.0 | 0.97 (0.58 – 1.62)  1.26 (0.72 – 2.23) | | 1.71 (0.79 – 3.69)  2.71 (1.14 – 6.44) | |  |  |
| Note: The RERI is a measure of interaction on the additive scale; it has a distribution of minus infinity to plus infinity with 0 as the equivalency point indicating no additive interaction. The prevalence odds ratio (POR) has a distribution of zero to plus infinity with 1 as the equivalency point. The potential confounders controlled for in the adjusted models include: age, sex, service branch, rank, active duty vs Guard/Reserve, special strata, and combat exposure scale. The analyses included 624 cases and 508 controls. Abbreviations: aRERI, relative excess risk due to interaction adjusted for measured confounding; CI, confidence interval; LR, logistic regression; PON1, paraoxonase-1. | | | | | | | | | |

|  |
| --- |

| **Table S18.** Interaction on the additive and multiplicative scales of hearing nerve agent alarms and *PON1* Q192R genotype on GWI including cases meeting the **CDC mild-to-moderate** **case definition excluding those meeting the Research** case definition | | | | | | | | | |
| --- | --- | --- | --- | --- | --- | --- | --- | --- | --- |
| Heard nerve agent alarms | | *PON1* Q192R genotype | | | | | |  | |
|  |  | QQ | | QR | | RR | | PORs for *PON1* Q192R genotypes within strata of alarms | |
|  |  | N  cases/ controls | POR (95%CI) | N  cases/ controls | POR (95%CI) | N  cases/ controls | POR (95%CI) | QR vs QQ | RR vs QQ |
|  | No | 77/130 | 1.0 | 92/120 | 1.29 (0.88-1.91) | 27/37 | 1.23 (0.70-2.18) | 1.29 (0.88-1.91) | 1.23 (0.70-2.18) |
|  | Yes | 122/104 | 1.98 (1.35-2.91) | 146/96 | 2.57 (1.75-3.76) | 57/21 | 4.58 (2.58-8.14) | 1.30 (0.90-1.87) | 2.31 (1.32-4.07) |
| POR (95%CI) for alarms within strata of genotypes | |  | 1.98 (1.35-2.91) |  | 1.98 (1.36-2.88) |  | 3.72 (1.84-7.53) |  |  |
| Additive scale: RERI (95%CI)  Unadjusted  Adjusted for confounders | | | 1.0  1.0 | 0.29 (-0.79 – 1.26)  0.50 (-0.49 – 1.43) | | 2.37 (0.33 – 5.69)  2.08 (0.46 – 4.92) | |  |  |
| Multiplicative scale: POR (95%CI) from LR interaction term  Unadjusted  Adjusted for confounders | | | 1.0  1.0 | 1.00 (0.59 – 1.71)  1.24 (0.69 – 2.24) | | 1.88 (0.84 – 4.19)  2.79 (1.14 – 6.82) | |  |  |
| Note: The RERI is a measure of interaction on the additive scale; it has a distribution of minus infinity to plus infinity with 0 as the equivalency point indicating no additive interaction. The prevalence odds ratio (POR) has a distribution of zero to plus infinity with 1 as the equivalency point. The potential confounders controlled for in the adjusted models include: age, sex, service branch, rank, active duty vs Guard/Reserve, special strata, and combat exposure scale. The analyses included 521 cases and 508 controls. Abbreviations: aRERI, relative excess risk due to interaction adjusted for measured confounding; CI, confidence interval; LR, logistic regression; PON1, paraoxonase-1. | | | | | | | | | |

| **Table S19.** Interaction on the additive and multiplicative scales of hearing nerve agent alarms and *PON1* Q192R genotype on GWI including cases meeting the **CDC Severe case definition excluding those meeting the Research** case definition | | | | | | | | | |
| --- | --- | --- | --- | --- | --- | --- | --- | --- | --- |
| Heard nerve agent alarms | | *PON1* Q192R genotype | | | | | |  | |
|  |  | QQ | | QR | | RR | | PORs for *PON1* Q192R genotypes within strata of alarms | |
|  |  | N  cases/ controls | POR (95%CI) | N  cases/ controls | POR (95%CI) | N  cases/ controls | POR (95%CI) | QR vs QQ | RR vs QQ |
|  | No | 13/130 | 1.0 | 14/120 | 1.17 (0.53-2.58) | 6/37 | 1.62 (0.58-4.56) | 1.17 (0.53-2.58) | 1.62 (0.58-4.56) |
|  | Yes | 31/104 | 2.98 (1.49-5.99) | 28/96 | 2.92 (1.44-5.93) | 11/21 | 5.24 (2.08-13.22) | 0.98 (0.55-1.75) | 1.76 (0.77-4.04) |
| POR (95%CI) for alarms within strata of genotypes | |  | 2.98 (1.49-5.99) |  | 2.50 (1.25-5.01) |  | 3.23 (1.04-10.00) |  |  |
| Additive scale: RERI (95%CI)  Unadjusted  Adjusted for confounders | | | 1.0  1.0 | -0.23 (-3.38 – 1.93)  0.53 (-1.70 – 1.95) | | 1.64 (-2.8 – 8.76)  2.11 (-0.85 – 8.95) | |  |  |
| Multiplicative scale: POR (95%CI) from LR interaction term  Unadjusted  Adjusted for confounders | | | 1.0  1.0 | 0.84 (0.31 – 2.25)  1.53 (0.51 – 4.63) | | 1.08 (0.29 – 4.09)  2.87 (0.58 – 14.10) | |  |  |
| Note: The RERI is a measure of interaction on the additive scale; it has a distribution of minus infinity to plus infinity with 0 as the equivalency point indicating no additive interaction. The prevalence odds ratio (POR) has a distribution of zero to plus infinity with 1 as the equivalency point. The potential confounders controlled for in the adjusted models include: age, sex, service branch, rank, active duty vs Guard/Reserve, special strata, and combat exposure scale. The analyses included 103 cases and 508 controls. Abbreviations: aRERI, relative excess risk due to interaction adjusted for measured confounding; CI, confidence interval; LR, logistic regression; PON1, paraoxonase-1. | | | | | | | | | |
|  | | | | | | | | | |

| **Table S20.** Interaction on the additive and multiplicative scales of hearing nerve agent alarms and *PON1* Q192R genotype on GWI including cases meeting the **Modified Kansas definition with no exclusions case definition excluding those meeting the Research** case definition | | | | | | | | | |
| --- | --- | --- | --- | --- | --- | --- | --- | --- | --- |
| Heard nerve agent alarms | | *PON1* Q192R genotype | | | | | |  | |
|  |  | QQ | | QR | | RR | | PORs for *PON1* Q192R genotypes within strata of alarms | |
|  |  | N  cases/ controls | POR (95%CI) | N  cases/ controls | POR (95%CI) | N  cases/ controls | POR (95%CI) | QR vs QQ | RR vs QQ |
|  | No | 75/130 | 1.0 | 105/120 | 1.52 (1.03-2.32) | 32/37 | 1.50 (0.86-2.60) | 1.52 (1.03-2.32) | 1.50 (0.86-2.60) |
|  | Yes | 151/104 | 2.52 (1.72-3.67) | 169/96 | 3.05 (2.09-4.46) | 70/21 | 5.78 (3.29-10.16 | 1.21 (0.85-1.73) | 2.30 (1.33-3.97) |
| POR (95%CI) for alarms within strata of genotypes | |  | 2.52 (1.72-3.67) |  | 2.01 (1.40-2.89) |  | 3.85 (1.95-7.60) |  |  |
| Additive scale: RERI (95%CI)  Unadjusted  Adjusted for confounders | | | 1.0  1.0 | 0.02 (-1.28 – 1.14)  0.27 (-0.91 – 1.32) | | 2.76 (0.28 – 6.83)  2.79 (0.68 – 6.67) | |  |  |
| Multiplicative scale: POR (95%CI) from LR interaction term  Unadjusted  Adjusted for confounders | | | 1.0  1.0 | 0.80 (0.47 – 1.35)  1.01 (0.55 – 1.82) | | 1.53 (0.70 – 3.33)  2.58 (1.06 – 6.26) | |  |  |
| Note: The RERI is a measure of interaction on the additive scale; it has a distribution of minus infinity to plus infinity with 0 as the equivalency point indicating no additive interaction. The prevalence odds ratio (POR) has a distribution of zero to plus infinity with 1 as the equivalency point. The potential confounders controlled for in the adjusted models include: age, sex, service branch, rank, active duty vs Guard/Reserve, special strata, and combat exposure scale. The analyses included 602 cases and 508 controls. Abbreviations: aRERI, relative excess risk due to interaction adjusted for measured confounding; CI, confidence interval; LR, logistic regression; PON1, paraoxonase-1. | | | | | | | | | |
|  | | | | | | | | | |

| **Table S21.** Interaction on the additive and multiplicative scales of hearing nerve agent alarms and *PON1* Q192R genotype on GWI including cases meeting the **Modified Kansas with exclusions case definition excluding those meeting the Research** case definition | | | | | | | | | |
| --- | --- | --- | --- | --- | --- | --- | --- | --- | --- |
| Heard nerve agent alarms | | *PON1* Q192R genotype | | | | | |  | |
|  |  | QQ | | QR | | RR | | PORs for *PON1* Q192R genotypes within strata of alarms | |
|  |  | N  cases/ controls | POR (95%CI) | N  cases/ controls | POR (95%CI) | N  cases/ controls | POR (95%CI) | QR vs QQ | RR vs QQ |
|  | No | 66/130 | 1.0 | 85/120 | 1.40 (0.93-2.09) | 26/37 | 1.38 (0.77-2.48) | 1.40 (0.93-2.09) | 1.38 (0.77-2.48) |
|  | Yes | 125/104 | 2.37 (1.60-3.51) | 145/96 | 2.98 (2.01-4.41) | 62/21 | 5.82 (3.27-10.35) | 1.26 (0.87-1.81) | 2.46 (1.40-4.30) |
| POR (95%CI) for alarms within strata of genotypes | |  | 2.37 (1.60-3.51) |  | 2.13 (1.46-3.12) |  | 4.20 (2.08-8.50) |  |  |
| Additive scale: RERI (95%CI)  Unadjusted  Adjusted for confounders | | | 1.0  1.0 | 0.21 (-1.06 – 1.34)  0.43 (-0.69 –­­­­­­ 1.47) | | 3.06 (0.56 – 7.28)  2.92 (0.85 – 6.82) | |  |  |
| Multiplicative scale: POR (95%CI) from LR interaction term  Unadjusted  Adjusted for confounders | | | 1.0  1.0 | 0.90 (0.52 – 1.56)  1.16 (0.62 – 2.16) | | 1.77 (0.79 – 3.98)  3.04 (1.22 – 7.60) | |  |  |
| Note: The RERI is a measure of interaction on the additive scale; it has a distribution of minus infinity to plus infinity with 0 as the equivalency point indicating no additive interaction. The prevalence odds ratio (POR) has a distribution of zero to plus infinity with 1 as the equivalency point. The potential confounders controlled for in the adjusted models include: age, sex, service branch, rank, active duty vs Guard/Reserve, special strata, and combat exposure scale. The analyses included 509 cases and 508 controls. Abbreviations: aRERI, relative excess risk due to interaction adjusted for measured confounding; CI, confidence interval; LR, logistic regression; PON1, paraoxonase-1. | | | | | | | | | |
|  | | | | | | | | | |

| **Table S22**. Test for heterogeneity of the GxE interaction over **age groups,** by GWI case definition controlling for confounding. | | | | | | | | | | | | | | | | | | | |
| --- | --- | --- | --- | --- | --- | --- | --- | --- | --- | --- | --- | --- | --- | --- | --- | --- | --- | --- | --- |
|  | PON1 Q192R genotype | Heard nerve agent alarms | Research | |  | CDC | |  | Kansas without exclusions | |  | | Kansas with exclusions | |  | | CDC Severe | | |
| Age group |  |  | Cases | Controls |  | Cases | Controls |  | Cases | Controls |  | | Cases | Controls |  | | Cases | | Controls |
| <45 yr | QQ | No | 24 | 88 |  | 23 | 88 |  | 23 | 88 |  | | 13 | 88 |  | | 9 | | 88 |
|  |  | Yes | 73 | 76 |  | 73 | 76 |  | 73 | 96 |  | | 45 | 76 |  | | 33 | | 76 |
|  | RR | No | 9 | 29 |  | 8 | 29 |  | 9 | 29 |  | | 7 | 29 |  | | 4 | | 29 |
|  |  | Yes | 53 | 15 |  | 50 | 15 |  | 50 | 15 |  | | 27 | 15 |  | | 26 | | 15 |
|  | Tests of GxE interaction: | | RERI = 8.4 (2.5-24.1) | |  | RERI = 3.2 (1.2-7.5) | |  | RERI = 4.5 (1.6-10.6) | |  | | RERI = 4.4 (1.5-10.6) | |  | | RERI = 6.9 (2.3-21.1) | | |
| ≥45 yr | QQ | No | 19 | 42 |  | 18 | 42 |  | 18 | 42 |  | | 12 | 42 |  | | 7 | | 42 |
|  |  | Yes | 56 | 28 |  | 54 | 28 |  | 54 | 28 |  | | 14 | 28 |  | | 34 | | 28 |
|  | RR | No | 9 | 8 |  | 9 | 8 |  | 9 | 8 |  | | 3 | 8 |  | | 3 | | 8 |
|  |  | Yes | 38 | 6 |  | 36 | 6 |  | 35 | 6 |  | | 13 | 6 |  | | 22 | | 6 |
|  | Tests of GxE interaction: | | RERI = 4.3 (-5.2-27.9) | |  | RERI = 3.0 (-2.6-16.1) | |  | RERI = 3.6 (-2.6-19.2) | |  | | RERI = 2.9 (-1.4-14.8) | |  | | RERI = 6.5 (-8.1-51.0) | | |
| Test of homogeneity of the GxE interaction over age* | | | *P* = 0.14 | |  | *P* = 0.11 | |  | *P* = 0.15 | |  | *P* = 0.45 | | | |  | | *P* = 0.18 | |
| Abbreviations: RERI, relative excess risk due to interaction; GxE, gene-environment. | | | | | | | | | | | | | | | | | | | |
| *Homogeneity of the GxE interaction over age was tested by significance of the logistic regression coefficient on the 3-way interaction of genotype x alarms x age with the given case definition of GWI. The QR subjects were excluded to compare the extreme genotypes QQ vs RR. | | | | | | | | | | | | | | | | | | | |
| All statistical analyses controlled for sex, service branch, rank, active duty vs Guard/Reserve, special strata, and combat exposure scale. | | | | | | | | | | | | | | | | | | | |

| **Table S23**. Test for heterogeneity of the GxE interaction over **sex,** by GWI case definition controlling for confounding. | | | | | | | | | | | | | | | | |
| --- | --- | --- | --- | --- | --- | --- | --- | --- | --- | --- | --- | --- | --- | --- | --- | --- |
|  | PON1 Q192R genotype | Heard nerve agent alarms | Research | |  | CDC | |  | Kansas without exclusions | |  | Kansas with exclusions | |  | CDC Severe | |
| Sex |  |  | Cases | Controls |  | Cases | Controls |  | Cases | Controls |  | Cases | Controls |  | Cases | Controls |
| Male | QQ | No | 33 | 127 |  | 31 | 127 |  | 31 | 127 |  | 18 | 127 |  | 13 | 127 |
|  |  | Yes | 103 | 94 |  | 101 | 94 |  | 101 | 94 |  | 46 | 94 |  | 53 | 94 |
|  | RR | No | 12 | 34 |  | 11 | 34 |  | 12 | 34 |  | 6 | 34 |  | 5 | 34 |
|  |  | Yes | 66 | 19 |  | 62 | 19 |  | 61 | 19 |  | 25 | 19 |  | 35 | 19 |
|  | Tests of GxE interaction: | | RERI = 7.8 (2.1-21.7) | |  | RERI = 2.9 (0.8-6.7) | |  | RERI = 4.0 (1.2-9.3) | |  | RERI = 3.5 (1.1-8.2) | |  | RERI = 6.1 (1.4-18.1) | |
| Female | QQ | No | 10 | 3 |  | 10 | 3 |  | 10 | 3 |  | 7 | 3 |  | 3 | 3 |
|  |  | Yes | 26 | 10 |  | 26 | 10 |  | 26 | 10 |  | 13 | 10 |  | 14 | 10 |
|  | RR | No | 6 | 3 |  | 6 | 3 |  | 6 | 3 |  | 4 | 3 |  | 2 | 3 |
|  |  | Yes | 25 | 2 |  | 24 | 2 |  | 24 | 2 |  | 15 | 2 |  | 13 | 2 |
|  | Tests of GxE interaction: | | RERI = 11.0 (0.9-157.3) | |  | RERI = 5.8 (0.5-71.3) | |  | RERI = 6.8 (0.2-85.6) | |  | RERI = 7.5 (-0.5-103.1) | |  | RERI = (inestimable) | |
| Test of homogeneity of the GxE interaction over sex* | | | *P* = 0.64 | |  | *P* = 0.55 | |  | *P* = 0.63 | |  | *P* = 0.52 | |  | *P* = 0.94 | |
| Abbreviations: RERI, relative excess risk due to interaction (95% confidence interval); GxE, gene-environment. | | | | | | | | | | | | | | | | |
| *Homogeneity of the GxE interaction over sex was tested by significance of the logistic regression coefficient on the 3-way interaction of genotype x alarms x sex with the given case definition of GWI. The QR subjects were excluded to compare the extreme genotypes QQ vs RR. | | | | | | | | | | | | | | | | |
| All statistical analyses controlled for age, service branch, rank, active duty vs Guard/Reserve, special strata, and combat exposure scale | | | | | | | | | | | | | | | | |

|  |  | | | | | | | | | | | | | | | | |  |
| --- | --- | --- | --- | --- | --- | --- | --- | --- | --- | --- | --- | --- | --- | --- | --- | --- | --- | --- |
|  | **Table S24. Estimation of sensitivity and specificity of GWI case definitions** | | | | | | | | | | | | | | | | |  |
|  |  | | A | B | C | D | E | F | G | H | I | J | K | L | M | N | O |  |
|  |  |  | *Sp_i_* | FP rate | Sample N of cases (*r*) | Sample N of non-cases (*r*) | Crude GWI Population Prevalence rate est. (%) | Population Prevalence rate of FP cases (%) | Population Prevalence rate of TP cases (%) | Population Prevalence rate of FN cases (%) | FN rate | ***Se_i_*** | **95% CI* of Se*_i_*** | | ***Sp_i_*** | **95% CI* of Sp*_i_*** | |  |
|  | Line No. | Case definition (*i*) | From the correction analysis (Table 2) | (1-A_i_) | From prevalence case-control sample (C*_i_*) | From prevalence case-control sample (D*_i_*) | From USMHS population estimate | (B_i_ · E_i_) | (A*_i_* · E*_i_*) or (E*_i_* - F*_i_*) | (G1 - G*_i_*) | (G1 - G*_i_*)/G1 | **(1 - I*_i_*)** | **LCL** | **UCL** | **(copied from A*_i_*)** | **LCL** | **UCL** |  |
|  | 1 | CDC | 0.819 | 0.181 | 615 | 292 | 41.7 | 7.5 | 34.2 | 0 | 0 | **1** |  |  | **0.819** | 0.775 | 0.855 |  |
|  | 2 | Kansas w/o excl | 0.839 | 0.161 | 599 | 292 | 39.0 | 6.3 | 32.7 | 0 | 0 | **1** |  |  | **0.839** | 0.798 | 0.875 |  |
|  | 3 | Kansas w excl | 0.786 | 0.214 | 413 | 292 | 25.6 | 5.5 | 20.1 | 14.0 | 0.411 | **0.589** | 0.552 | 0.625 | **0.786** | 0.741 | 0.825 |  |
|  | 4 | Research | 1 | 0 | 281 | 292 | 13.6 | 0 | 13.6 | 20.6 | 0.602 | **0.398** | 0.362 | 0.435 | **1** |  |  |  |
|  | 5 | CDC Severe | 1 | 0 | 199 | 292 | 10.6 | 0 | 10.6 | 23.6 | 0.690 | **0.310** | 0.276 | 0.347 | **1** |  |  |  |
|  |  |  |  | | *n* = C*_i_*/J_i_ | *n* = D*_i_*/M*_i_* |  |  |  |  |  | *Binomial-based exact mid-P CIs from SAS macro: | | | | | |  |
|  |  |  | Kansas w excl | | 701 | 357 | CDC |  |  |  |  | From the calculations at the left, enter *r* and *n* | | | | | |  |
|  |  |  | Research | | 706 | 348 | Kansas w/o excl | |  |  |  | (e.g., for CI of Se of Research enter *r* = 281, *n* = 706 | | | | | |  |
|  |  |  | CDC Severe | | 641 | 372 | Kansas w excl | |  |  |  | and for CI of Sp of CDC enter *r* = 292, *n* = 357). | | | | | |  |
|  |  |  |  | |  |  |  |  |  |  |  | CI of Se uses *r* of cases, CI of Sp uses *r* of non-cases. | | | | | |  |
|  |  | | | | | | | | |  | https://www.lexjansen.com/sesug/2015/103_Final_PDF.pdf | | | | | | |  |
|  | Abbreviations: Se, sensitivity; Sp, specificity; FP, false positive; FN, false negative; TP, true positive; TN, true negative; w excl, with exclusions; w/o excl, without exclusions. | | | | | | | | | | | | | | | | |  |

**References**

1. Haley RW, Kurt TL, Hom J: Is there a Gulf War Syndrome? Searching for syndromes by factor analysis of symptoms. JAMA 1997, 277(3):215-222.

2. Fukuda K, Nisenbaum R, Stewart G, Thompson WW, Robin L, Washko RM, Noah DL, Barrett DH, Randall B, Herwaldt BL et al: Chronic multisymptom illness affecting Air Force veterans of the Gulf War. J Am Med Assoc 1998, 280(11):981-988.

3. Steele L: Prevalence and patterns of Gulf War illness in Kansas veterans: association of symptoms with characteristics of person, place, and time of military service. Am J Epidemiol 2000, 152(10):992-1002.

4. Dursa E, Barth S, Porter B, Schneiderman A: Gulf War illness in the 1991 Gulf War and Gulf Era Veteran population: An application of the Centers for Disease Control and Prevention and Kansas case definitions to historical data. J Milit Vet Health 2018, 26:43-50.

5. Coughlin SS, McNeil RB, Provenzale DT, Dursa EK, Thomas CM: Method Issues in Epidemiological Studies of Medically Unexplained Symptom-based Conditions in Veterans. J Milit Vet Health 2013, 21(2):4-10.

6. Brewer KL, Mainhart A, Meggs WJ: Double-blinded placebo-controlled cross-over pilot trial of naltrexone to treat Gulf War illness. Fatigue Biomed Health Behav 2018, 6(3):132-140.

7. Hu LT, Bentler PM: Cutoff Criteria for Fit Indexes in Covariance Structure Analysis: Conventional Criteria Versus New Alternatives. Struct Equat Model 1999, 6(1):1-55.

8. Haley RW, Luk GD, Petty F: Use of structural equation modeling to test the construct validity of a case definition of Gulf War syndrome: invariance over developmental and validation samples, service branches and publicity. Psychiatry Res 2001, 102(2):175-200.

9. Iannacchione VG, Dever JA, Bann CM, Considine KA, Creel D, Carson CP, et al.: Validation of a research case definition of Gulf War illness in the 1991 US military population. Neuroepidemiology 2011, 37(2):129-140.
